# The Spatial‐Temporal Alternative Splicing Profile Reveals the Functional Diversity of FXR1 Isoforms in Myogenesis

**DOI:** 10.1002/advs.202405157

**Published:** 2024-11-05

**Authors:** Wei Wang, Xinhao Fan, Weiwei Liu, Yuxin Huang, Shuhong Zhao, Yalan Yang, Zhonglin Tang

**Affiliations:** ^1^ Kunpeng Institute of Modern Agriculture at Foshan Agricultural Genomics Institute Chinese Academy of Agricultural Sciences Foshan 528226 China; ^2^ Key Laboratory of Agricultural Animal Genetics Breeding and Reproduction of Ministry of Education and Key Lab of Swine Genetics and Breeding of Ministry of Agriculture and Rural Affairs Huazhong Agricultural University Wuhan 430070 China; ^3^ Shenzhen Branch Guangdong Laboratory for Lingnan Modern Agriculture Key Laboratory of Livestock and Poultry Multi‐Omics of MARA Agricultural Genomics Institute at Shenzhen Chinese Academy of Agricultural Sciences Shenzhen 518124 China; ^4^ Guangxi Key Laboratory of Animal Breeding Disease Control and Prevention College of Animal Science & Technology Guangxi University Nanning 530004 China

**Keywords:** alternative splicing, FXR1, myogenesis, muscle regeneration, RBM24

## Abstract

Alternative splicing (AS) is a fundamental mechanism contributing to proteome diversity, yet its comprehensive landscape and regulatory dynamics during skeletal muscle development remain largely unexplored. Here, the temporal AS profiles are investigated during myogenesis in five vertebrates, conducting comprehensive profiling across 27 developmental stages in skeletal muscle and encompassing ten tissues in adult pigs. The analysis reveals a pervasive and evolutionarily conserved pattern of alternative exon usage throughout myogenic differentiation, with hundreds of skipped exons (SEs) showing developmental regulation, particularly within skeletal muscle. Notably, this study identifies a muscle‐specific SE (exon 15) within the *Fxr1* gene, whose AS generates two dynamically expressed isoforms with distinct functions: the isoform without exon 15 (*Fxr1^E15^* ^−^) regulates myoblasts proliferation, while the isoform incorporating exon 15 (*Fxr1^E15+^*) promotes myogenic differentiation and fusion. Transcriptome analysis suggests that specifically knocking‐down *Fxr1^E15+^* isoform in myoblasts modulates differentiation by influencing gene expression and splicing of specific targets. The increased inclusion of exon 15 during differentiation is mediated by the binding of *Rbm24* to the intron. Furthermore, in vivo experiments indicate that the *Fxr1^E15+^* isoform facilitates muscle regeneration. Collectively, these findings provide a comprehensive resource for AS studies in skeletal muscle development, underscoring the diverse functions and regulatory mechanisms governing distinct *Fxr1* isoforms in myogenesis.

## Graphical abstract

Conserved alternative splicing (AS) transitions are observed during myogenic differentiation across five species, with dynamic splicing of skipped exons (SEs) during development that specifically occurs in skeletal muscle. This study provides a valuable resource for investigating AS in skeletal muscle, particularly highlighting the regulation of a muscle‐specific SE (exon15) within *Fxr1* by *Rbm24*, resulting in two isoforms with distinct functions.

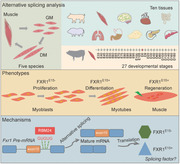

## 1 Introduction

Alternative splicing (AS) is a prevalent post‐transcriptional regulation, more than 95% of multi‐exon genes in the mammalian genome are alternatively spliced to form multiple transcripts. Different transcripts generated from the same gene often encoding proteins with differing or opposing function,^[^ ^1^ ^]^ contributing significantly to the protein diversity.^[^ ^2^ ^]^ The process of AS relies on the core spliceosome machinery while being finely tuned by *trans*‐acting splicing factors, such as SR^[^ ^3^, ^4^ ^]^ and hnRNP protein families,^[^ ^5^, ^6^ ^]^ and *cis*‐regulatory elements present in alternative exons and adjacent introns.^[^ ^7^ ^]^ AS frequently manifests in a cell‐type or tissue‐specific manner, playing important roles in cell specification, tissue development, and the maintenance of organ function.^[^ ^8^ ^]^ Compared with other tissues, an enrichment of tissue‐specific AS was observed in skeletal muscle,^[^ ^9^ ^]^ suggesting its critical role in skeletal muscle development.

Skeletal muscle is the most abundant tissue in the mammalian body and plays a pivotal role in regulating body metabolism and homeostasis.^[^ ^10^ ^]^ Myogenesis typically commences during embryonic development, where a group of undifferentiated myoblasts undergo a series of intricate molecular signaling events that guide their differentiation into mature, contractile muscle fibers.^[^ ^11^ ^]^ The regulation of myogenesis by the myogenic regulatory factors (MRFs) has been firmly established.^[^ ^12^, ^13^ ^]^ AS is recognized as a high dynamic and evolutionarily conserved process throughout myogenic differentiation.^[^ ^14^ ^]^ Using splicing‐sensitive microarray analysis, about one hundred of AS events were observed undergoing transitions during C2C12 differentiation.^[^ ^14^ ^]^ Meanwhile, many AS regulators, such as *Rbm24*,^[^ ^15^, ^16^, ^17^, ^18^ ^]^ *QKI*,^[^ ^19^ ^]^ and *Rbfox2*,^[^ ^20^, ^21^ ^]^ could regulate myogenesis and muscle regeneration though mediating muscle‐specific AS. For example, 30% of splicing transitions during myogenesis were regulated by *Rbfox2*, which coordinate AS of *Mef2d* and *Rock2* to control myoblast fusion.^[^ ^20^ ^]^ However, the comprehensive landscape, regulatory dynamics and molecular mechanism of AS during skeletal muscle development are still largely unclear.

The transcriptome sequencing greatly expanded our knowledge of the molecular regulation in skeletal muscle development at the gene expression level.^[^ ^22^, ^23^, ^24^ ^]^ In this study, using large‐scale transcriptome datasets collected from differentiating myoblasts, skeletal muscle and non‐muscle tissues, we observed thousands of conserved AS transitions during myogenic differentiation across human, mouse and three livestock. Hundreds of differentiation‐associated skipped exons (SEs) were dynamically spliced during skeletal muscle development, specifically included or excluded in skeletal muscle, including the exon 15 of *Fxr1*, a gene well‐known for its involvement in vascular smooth muscle cells, skeletal muscle development and diseases.^[^ ^25^, ^26^, ^27^, ^28^ ^]^ Multiple exons of *Fxr1* are dynamic spliced during myogenesis,^[^ ^25^ ^]^ with the AS of exon 15 generating muscle‐specific *Fxr1* isoforms.^[^ ^29^ ^]^ We investigated the functions and regulatory mechanisms of various *Fxr1* isoforms in myogenesis and muscle regeneration induced by exon 15 splicing. Overall, our study provides a rich resource for the study of AS in skeletal muscle and highlight the important of muscle‐specific AS in myogenesis gene regulation.

## 2 Results

### 2.1 Extensive and Conserved Alternative Exon Usages During Myogenic Differentiation

**Figure 1:**
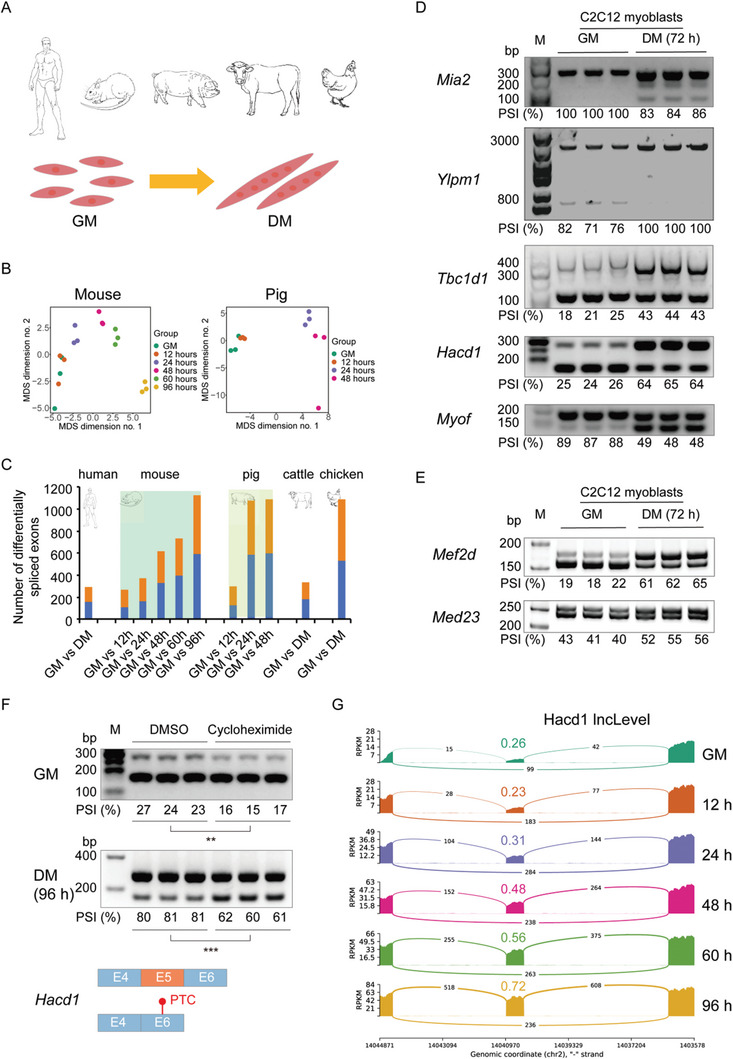
Conservation analysis of AS events across multiple species in proliferation and differentiation of myoblasts. A) Schematic representation of the underlying RNA‐seq dataset for the five species (human, mouse, pig, cattle and chicken), along with the morphological changes from myoblasts to myotubes during the proliferation stage (GM, growth medium) and differentiation stage (DM, differentiation medium). The human and animal sketches were adopted from the SKMATLAS database (https://skmatlas.cn/). B) MDS plot showing the global view of dynamic PSI values during the GM and DM phases in mouse and pig, colored by differentiation hours. C) Number of DASEs in myoblasts of the five species. Blue and orange colors represent upregulated and downregulated SEs, respectively. D) Semi‐quantitative RT‐PCR analyses of splicing changes of five representative DASEs between GM and DM (72 h) in C2C12 myoblasts (n = 3). E) Polyacrylamide gel electrophoresis analyses of splicing changes of two representative microexons between GM and DM (72 h) in C2C12 myoblasts (n = 3). F) Semi‐quantitative RT‐PCR analyses of splicing changes of the *Hacd1* NMD‐SE treating GM and DM (96 h) C2C12 myoblasts and myotubes with CHX (cycloheximide) or vehicle control (DMSO) for 6 h (n = 3). PTC, premature termination codon. In D‐F, the PSI values are displayed below each gel image. G) Sashimi plot based on the RNA‐seq data showing splicing changes of the *Hacd1* NMD‐SE during C2C12 myoblasts proliferation (GM) and differentiation (DM; 12, 24, 48, 60, and 96 h). PSI value was given according to the ratio of the long form on total form present (short form and long form) to characterize inclusion of exon. *P‐*values were calculated using Student's t‐test. **P < 0.01, ***P < 0.001.

During myogenic differentiation, the cell morphology changes from flat, fusiform or star‐shaped mononucleated cells into elongated and fused multinucleated MyHC‐positive cells (**Figure** **1**A). To analysis AS changes during myogenic differentiation, we collected 48 RNA‐seq datasets^[^ ^30^, ^31^, ^32^, ^33^ ^]^ from proliferating (growth medium, GM) and differentiating myoblasts (differentiation medium, DM) in five species (human, mouse, pig, cattle and chicken). In mouse and pig, the differentiating myoblasts were collected at multiple time points (Figure 1A). Using rMATs package,^[^ ^34^ ^]^ we identified five types of AS events, including skipped exon (SE), mutually exclusive exon (MXE), alternative 5’ splice site (A5SS), alternative 3’ splice site (A3SS), and retained intron (RI) (Figure S1A, Supporting Information). Given SE is the most prevalent and most well‐characterized type of AS events in mammalian transcriptomes,^[^ ^35^ ^]^ we focused subsequent analyses on SE events. The multidimensional scaling (MDS) analysis based on the PSI values of detected SEs showed distinct patterns between early stages (GM and 12 h) and later stages (after 24 h) in pig and mouse (Figure 1B), suggesting significant splicing transitions during myogenic differentiation.

Next, we identified differentially alternatively spliced SEs between the GM and DM phases, which are considered as differentiation‐associated SEs (DASEs). A total of 291, 1839, 1789, 337 and 1092 DASEs were identified in human, mouse, pig, cattle, and chicken, respectively (Figure 1C; Figure S1B and Table S1, Supporting Information). As expected, the number of DASEs was increased with the differentiation process in mouse and pig. Among these DASEs, 1212 of them were conservedly differentially spliced in at least two species, suggesting a widespread and conserved evolutionarily pattern of alternative exon usage throughout myogenic differentiation.

Gene Ontology (GO) analysis suggested that genes with DASEs, such as *Hacd1*,^[^ ^36^ ^]^ *Myof*,^[^ ^37^ ^]^ *Mia2*, *Tbc1d1* ^[^ ^38^ ^]^ and *Ylpm1*, were significantly involved in muscle system process and muscle cell differentiation (Figure S1C, Supporting Information). The splicing transitions of selected DASEs, which exhibit distinct characteristics in exon length, structure and splicing pattern, were further confirmed by semi‐quantitative RT‐PCR (Figure 1D). Microexons are a special class of very short (3‐27 nt) and predominantly frame‐preserving cassette exon.^[^ ^39^, ^40^, ^41^ ^]^ In this study, we found that 20 DASEs were conserved microexons (Table S2, Supporting Information), such as those in transcription regulators *Med23* ^[^ ^42^ ^]^ and *Mef2d*. The splicing changes of these two microexons were further validated by polyacrylamide gel electrophoresis in skeletal muscle cells of mouse and pig (Figure 1E; Figure S1D, Supporting Information). Meanwhile, we observed 89 conserved DASEs (Table S3, Supporting Information) that potentially cause nonsense‐mediated mRNA decay (NMD), such as the skipping exon 5 (122 nt) of *Hacd1*. We treated the C2C12 myoblasts with cycloheximide (CHX) to suppress protein translation and NMD, and found that the PSI value of *Hacd1* exon 5 was significantly repressed at both the GM and DM stages after cycloheximide treatment (Figure 1F). What's more, the RNA‐seq and semi‐quantitative RT‐PCR both confirmed that the PSI values for the exon 5 demonstrated a continuous increase during myogenic differentiation (Figure 1G; Figure S1E, Supporting Information). The results indicate that AS of myogenesis genes regulates protein isoforms and/or gene expression levels during differentiation.

### 2.2 The Developmental AS Atlas of Skeletal Muscle

**Figure 2:**
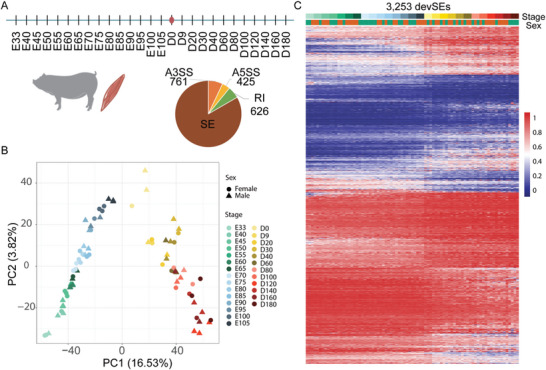
The developmental AS atlas of skeletal muscle in pigs. A) Identification of AS events in skeletal muscle across 27 developmental stages in pigs. E, embryonic stage; D, postnatal days. B) PCA analysis based on the PSI values showing the global splicing pattern across 27 developmental stages in pigs. Colored by developmental stage and shaped by sex. C) Heatmap showing the splicing changes of devSEs during skeletal muscle development.

Our previous study generated the transcriptome map of skeletal muscle across 27 developmental stages in pigs,^[^ ^24^ ^]^ which is a rich resource to profile the developmental AS atlas of skeletal muscle. After events calling and stringent filtering, we identified 10 883 high‐confident AS events, including 9071 SEs in 4218 genes (**Figure** **2**A). The MDS analysis based on PSI values of SE events suggested that skeletal muscles were ordered by developmental stage in a characteristic inverted V shape (Figure 2B), similar to the result based on genome‐wide gene expression,^[^ ^24^ ^]^ suggesting AS programs that steadily diverge during development, and coordination between gene expression and exon splicing.

To elucidate the dynamics and regulation of AS during skeletal muscle development, we identified developmentally dynamic SEs (devSEs) which showed significant changes of inclusion or exclusion frequencies through development using generalized linear models with quasi‐binomial distribution (quasi‐likelihood ratio test) as previously reported.^[^ ^35^ ^]^ We found 35.86% of total SEs (n = 3253) were dynamically spliced during development (adjusted *P*‐value ≤ 0.05, Figure 2C; Table S4, Supporting Information). Compared to the non‐devSEs, more devSEs are microexons (4.43% vs 2.20%). The lengths of devSEs (median = 104 bp) were significantly shorted than those of non‐devSEs (median = 111 bp) (*P* = 1.44e‐6), while no differences were observed in the GC content between devSEs and non‐devSEs (*P* = 0.9; Figure S2, Supporting Information). GO analysis suggested that genes with devSEs were significantly enriched in functional categories related to protein phosphorylation (FDR = 0.0049) and mRNA processing (FDR = 0.005). Meanwhile, we found that ≈80% (3339/4218) of devSEs related genes were dynamic expressed during skeletal muscle development. These results suggested that the dynamical expression and splicing were concordant during skeletal muscle development. We also found that 452 devSEs of them was differentially spliced between GM and DM phases (Table S4, Supporting Information), including SEs in myocyte enhancer factors *MEF2A* and *MEF2D*.

### 2.3 Conserved Exons Relate to Differentiation show Specific Splicing Pattern in Skeletal Muscle

**Figure 3:**
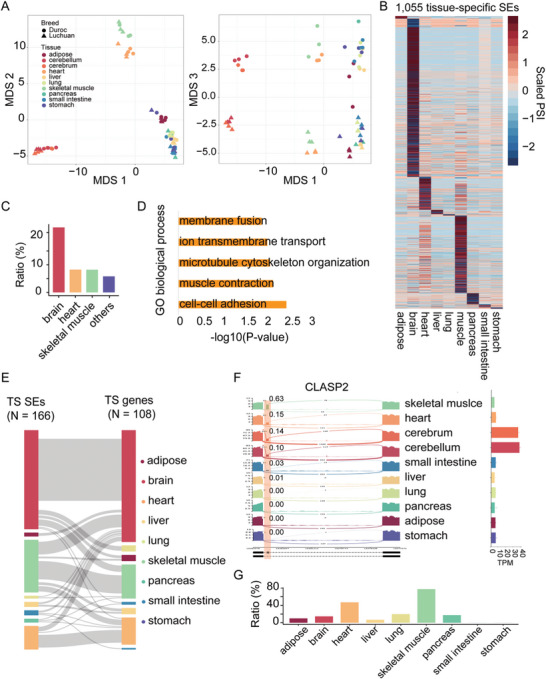
Global splicing analysis in different tissues of pigs. A) MDS analysis based on the PSI values showing the global splicing pattern across tissues in Luchuan and Duroc pigs. Colored by tissues and shaped by breeds. B) Heatmap showing the splicing pattern of tissue‐specific SEs. C) The ratio of microexons in each tissue. The ratio was calculated by dividing the number of microexons by the number of tissue‐specific SEs in each tissue. D) GO analysis of genes with skeletal muscle‐specific SEs. E) Sankey plot showing the overlap between genes with tissue‐specific SEs and tissues‐specific expressed genes. F) Sashimi plot showing a skeletal muscle‐specific SE in the *CLASP2* gene (left panel), which is a brain specific expressed gene (right panel). G) The ratio of devSEs in each tissue. The ratio was calculated by dividing the number of devSEs by the number of tissue‐specific exons in each tissue.

To analysis whether DASEs are specifically spliced in skeletal muscle, we profiled the AS pattern in 57 RNAs‐seq datasets of ten tissues in two pig breeds (Luchuan and Duroc).^[^ ^43^ ^]^ For the 7861 SEs discovered in tissues, 74.51% of them (5857/7861) were also undergone AS in developing skeletal muscles. MDS analysis based on the PSI values revealed that the first and second components separated the brain and skeletal muscle/heart from the other tissues, while the third component separated the Luchuan and Duroc pigs (**Figure** **3**A), suggesting that tissue type is the major contributor of AS.

Compared with AS events in other tissues, AS has disproportionate roles in the brain and muscle.^[^ ^35^ ^]^ We identified 1055 tissue‐specific SEs (modH < 2; Table S5, Supporting Information), which are specifically included or skipped in 744 genes. The most of them were specifically spliced in brain (n = 570), skeletal muscle (n = 280) and heart (n = 119) (Figure 3B). We found an excess of microexons with tissue‐specific splicing in the brain when compared with the other organs (Figure 3C), confirming the important role of microexons in the brain. GO enrichment analysis revealed that genes with muscle‐specific SEs, such as those in TPM1, TPM2 and TPM3, were enriched in cell‐cell adhesion and muscle contraction (Figure 3D).

We next analysis the expression pattern of genes with tissue‐specific SEs in various tissues. We found most tissue‐specific SEs are occurred in genes with ubiquitously expressed patterns across tissues. Only 108 of them (108/744) were tissue‐specific expressed, most of them were specifically spliced and expressed at the same tissue. However, we also observed that some tissue‐specific SEs are specifically expressed in other tissues (Figure 3E). For example, 14 genes with skeletal muscle‐specific SEs are specifically expressed in brain, such as the *CLASP2* gene (Figure 3F; Figure S3A, Supporting Information).

We explored whether the skeletal muscle‐specific SEs were differentially spliced during myogenic differentiation and dynamically regulated during skeletal muscle development. Interestingly, most of the skeletal muscle‐specific SEs were devSEs (217/280 = 78%; Table S5, Supporting Information), this enrichment was also observed in heart (56/119 = 47%) (Figure 3G). For the 217 skeletal muscle‐specific devSEs, 49 of them were conservedly differentially spliced between DM and GM phases across species (Table S6, Supporting Information), such as exons in genes *TPM3*, *FXR1*, *CAMK2D* and *BIN1*. We further analyzed the splicing pattern of these 49 DASEs across various tissues. As shown in Figure S3B (Supporting Information), the majority of these DASEs (42/49) exhibited higher inclusion levels in the DM phase compared to the GM phase. Specifically, the inclusion levels of DM‐upregulated SEs in skeletal muscle are significantly higher than those in other tissues, while the inclusion levels of DM‐downregulated SEs in skeletal muscle are notably reduced in comparison to other tissues. These results highlight that dozens of conserved DASEs are specifically spliced in skeletal muscle and dynamically regulated during development, implying their important roles in myogenesis.

### 2.4 AS of Conserved *Fxr1* Exon 15 Generates Isoforms with Distinct Expression Patterns

**Figure 4:**
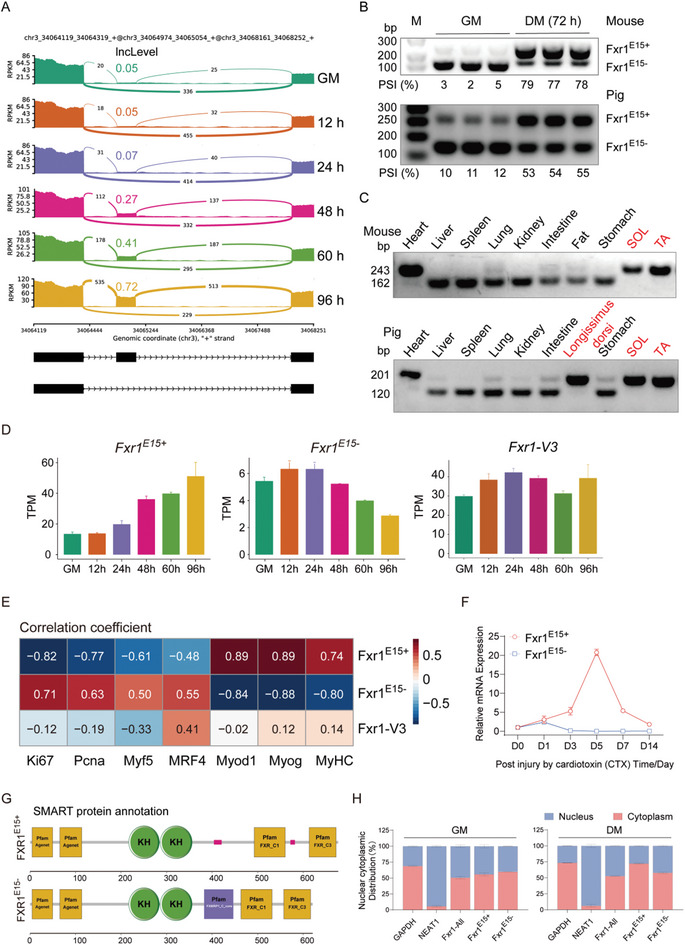
AS of *Fxr1* exon 15 generates isoforms with different expression patterns. A) Sashimi plot showing AS of *Fxr1* exon 15 during C2C12 myoblasts proliferation (GM) and differentiation (DM; 12, 24, 48, 60, and 96 h). B,C) Semi‐quantitative RT‐PCR showing splicing changes of *Fxr1* exon 15 during myoblasts differentiation (72 h) in mouse C2C12 myoblasts and pig skeletal muscle satellite cells (B) (n = 3) and in different tissues of mouse and pigs (C). D) The expression trend of *Fxr1^E15+^, Fxr1^E15^* ^−^ and *Fxr1‐V3* at different differentiation points of myoblasts. E) Heatmap showing the expression correlation of *Fxr1^E15+^* and *Fxr1^E15^* ^−^ with myoblast proliferation and differentiation markers based on the RNA‐seq data in C2C12 myoblasts. The values and color represent the correlation coefficient. F) qRT‐PCR assay to verify the expression trend of *Fxr1^E15+^* and *Fxr1^E15^* ^−^ during muscle regeneration. Mouse tibialis anterior (TA) muscles were collected on day 0 (uninjured) and on days 1, 3, 5, 7 and 14 after CTX (cardiotoxin) injection. The results are represented as the means ± SD, n = 3–5. G) The protein domains of the two isoforms of FXR1 predicted by Simple Modular Architecture Research Tool (SMART) website (http://smart.embl‐heidelberg.de/). H) Determination of *Fxr1* localization by nuclear‐cytoplasmic fractionation in DM and GM (n = 3). The results are represented as the means ± SD. The *Fxr1* isoform that includes exon 15 is named *Fxr1^E15+^*, and the isoform that does not include exon 15 is named *Fxr1^E15^* ^−^.

Previous studies have highlighted the crucial role of *Fxr1* in skeletal muscle development and diseases.^[^ ^25^, ^26^ ^]^ Consistent with previous findings in mice,^[^ ^25^ ^]^ the PSI values for *Fxr1* exon 15 demonstrated a continuous increase from the GM phase to the DM phase during myoblast differentiation (**Figure** **4**A; Figure S4A, Supporting Information), and the exon 15 showed specific inclusion in skeletal muscle and heart tissues (Figure 4C). Same splicing patterns are also observed in pig skeletal muscle cells and tissues (Figure 4B,C; Figure S4B, Supporting Information). In addition, we found that the inclusion level of exon 15 continuously increased during prenatal skeletal muscle development in pigs, and maintained a high level after birth (Figure S4C, Supporting Information).

Expression analysis using publicly available transcriptome data verified the specific expression of the *Fxr1* gene in skeletal muscle (Figure S4D, Supporting Information). Among the multiple isoforms of the *Fxr1* gene, three isoforms (V1‐V3) with distinct intron‐exon structures were predominantly expressed in myoblasts (Figure S5A,B, Supporting Information), which were validated by sanger sequencing (Figure S5C, Supporting Information). Isoform V1 (*Fxr1^E15+^*), which contains exon 15, displayed an increasing expression pattern with the progression of myoblast differentiation. In contrast, the exon 15 skipped isoform (V2, *Fxr1^E15^* ^−^) exhibited the opposite trend (Figure 4D). No significant expression change was observed in isoform V3 (*Fxr1‐V3*) during differentiation (Figure 4D), so *Fxr1‐V3* was not included for further analysis.

The expression of the *Fxr1* gene, *Fxr1^E15+^* and *Fxr1^E15^* ^−^ during myogenesis were further validated through qRT‐PCR (Figure S5D,E, Supporting Information) using the primers shown in Figure S5F (Supporting Information). We observed a highly positive correlation between the expression of *Fxr1^E15^* ^−^ and proliferation markers (*Ki67* and *PCNA*), while the expression of *Fxr1^E15+^* was highly correlated with those of differentiation markers (*Myod1*, *Myog* and *MyHC*) (Figure 4E; Figure S5G, Supporting Information). Furthermore, we analyzed the expression of these two isoforms during muscle regeneration in mice and observed a dramatic increase in the expression of *Fxr1^E15+^* during the initial 5 days after injury, followed by a decrease at day 7 (Figure 4F; Figure S5H, Supporting Information).

Additionally, we predicted the protein domains of *Fxr1* ^*E15+*^ and *Fxr1* ^*E15−*^ using the SMART website,^[^ ^44^, ^45^ ^]^ and observed that the inclusion of exon 15 may lead to the deletion of one FXR‐ligand binding domain (Figure 4G), which alters the intrinsically disordered domain and protein folding. Subcellular localization analysis, based on chromatin fractionation experiments, revealed the distribution of both isoforms and the *Fxr1* gene in both the cytoplasm and nucleus (Figure 4H; Figure S5I, Supporting Information). The above results suggesting the AS of *Fxr1* exon 15 might generate different proteins with distinct functions in myogenesis.

### 2.5 Two Isoforms of *Fxr1* show Different Functions in Myoblast Proliferation and Differentiation

**Figure 5:**
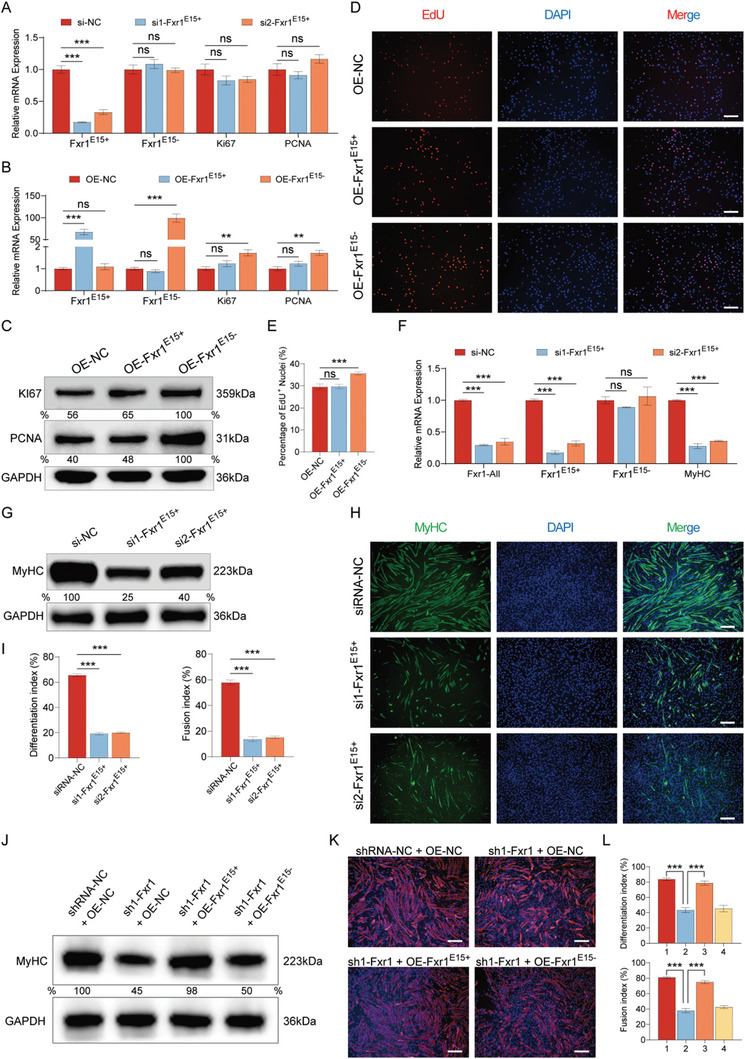
*Fxr1* isoforms act different functions in myoblast proliferation and differentiation. A) qRT‐PCR assay to verify the expression of *Fxr1^E15+^*, *Fxr1^E15^* ^−^ and myoblast proliferation markers after inhibiting the expression of *Fxr1^E15+^*. B) qRT‐PCR assay to verify the expression of myoblast proliferation marker genes after overexpression of *Fxr1^E15+^* and *Fxr1^E15^* ^−^. C) Western blot analysis of Ki67 and PCNA protein expression levels after overexpression of the two isoforms. D) EdU assay was carried out after transfection for 24 h. Cells undergoing DNA replication were stained by EdU (red) and cell nuclei were stained with DAPI (blue). Scale bar, 200 µm. E) Percentage of EdU^+^ nuclei were quantitated with ImageJ software. F) qRT‐PCR analysis of Fxr1‐All (all transcripts of *Fxr1*) and two isoforms of *Fxr1* mRNA expression after knocking‐down *Fxr1^E15+^*. The myogenic marker (*MyHC*) was also be detected. G) Western blot analysis of MyHC protein expression levels after transfecting siRNAs (si1 and si2) targeting *Fxr1^E15+^* (siFxr1‐exon 15). H) Immunofluorescence staining of C2C12 myoblasts differentiated for 3 days. Myotubes were labeled with MyHC (green) and cell nuclei were counterstained with DAPI (blue). Scale bar, 200 µm. I) Differentiation and fusion indexes of Figure 5H were quantitated with ImageJ software. J) Western blot analysis of MyHC expression after transfection of *Fxr1^E15+^* and *Fxr1^E15^* ^−^ overexpression plasmids into the FXR1‐knockdown cell line. K) Immunofluorescence staining of myoblasts differentiated for 3 days. Myotubes were labeled with MyHC (red) and cell nuclei were counterstained with DAPI (blue). Scale bar, 200 µm. L) Differentiation index and fusion index of Figure 5K were quantitated with ImageJ software. The numbers 1, 2, 3, and 4 represent the shRNA‐NC + OE‐NC, sh1‐Fxr1 + OE‐NC, sh1‐Fxr1 + OE‐Fxr1^E15+^ and sh1‐Fxr1 + OE‐Fxr1^E15−^ groups, respectively. The relative protein levels were normalized to those of the control GAPDH. The results are represented as the means ± SD. All data were obtained from at least three independent experiments. *P‐*values were calculated using Student's t‐test. **P < 0.01, ***P < 0.001. NC, negative control; ns indicates statistical non‐significance.

Considering the distinct expression patterns of *Fxr1^E15+^* and *Fxr1^E15^* ^−^, we hypothesized that they might play different roles in myogenesis. We designed two siRNAs targeting exon 15 of *Fxr1* to specifically knock‐down the *Fxr1^E15+^* transcript. qRT‐PCR results suggested these two siRNAs significantly inhibited the expression of *Fxr1^E15+^* with no effect on the expression of *Fxr1^E15^* ^−^ and proliferation markers *Ki67* and *PCNA* (**Figure** **5**A). Meanwhile, we constructed vectors that driving the expression of *Fxr1^E15+^* and *Fxr1^E15^* ^−^, and placed FLAG and HA tags on them, respectively (Figure S6A, Supporting Information), the western blot results showed that we had successfully overexpressed these two isoforms (Figure S6B, Supporting Information). After transfecting them into C2C12 myoblasts, the expression levels of *Ki67* and *PCNA* significantly increased when overexpression of *Fxr1^E15^* ^−^, whereas no changes were observed after *Fxr1^E15+^* overexpression (Figure 5B,C). The EdU proliferation assay was conducted to assess the proliferation index of C2C12 myoblasts. The proportion of EdU‐positive cells results indicated that only when *Fxr1^E15^* ^−^ was overexpressed, the proliferation rate of C2C12 myoblasts was accelerated (Figure 5D,E). These findings indicate that the *Fxr1^E15^* ^−^ isoform enhances myoblast proliferation, whereas *Fxr1^E15+^* does not affect cell proliferation.

To explore the role of *Fxr1* isoforms in myogenic differentiation, the two siRNAs targeting *Fxr1^E15+^* were transfected into C2C12 myoblasts and placed in DM for 72 h. The qRT‐PCR and western blot results indicated that we successfully reduced the expression of *Fxr1^E15+^* while having no impact on the expression of *Fxr1^E15^* ^−^ (Figure 5F; Figure S6D, Supporting Information). In addition, as a result of the downregulated expression of the *Fxr1^E15+^* transcript, the expression of the *Fxr1* gene decreased (Figure 5F). Furthermore, the expression of *Myod1*, *Myog* and *MyHC* significantly decreased at both RNA (Figure 5F; Figure S6C, Supporting Information) and protein levels (Figure 5G; Figure S6E, Supporting Information). The cell immunofluorescence results showed that following the specific knockdown of *Fxr1^E15+^*, cell differentiation capability decreased, manifesting as a reduced number of myotubes and a decreased number of nuclei within the myotubes (Figure 5H,I).

Depletion of *FXR1* has been shown to limit proliferative abilities in myoblasts.^[^ ^46^ ^]^ We constructed a *Fxr1* knockdown cell line to inactivate the expression of all *Fxr1* isoforms by packaging two different shRNA lentiviral vectors targeting two constitutive exons (exon 9 and exon 10). qRT‐PCR performed on cells infected with *Fxr1* shRNA lentivirus resulted in a significant reduction in *Fxr1* mRNA compared to shControl‐infected cells (Figure S6I, Supporting Information). Moreover, *Fxr1* knockdown significantly decreased the expression of the proliferation markers *Ki67*, *PCNA* and myogenic marker *MyHC* in myoblasts, as demonstrated by qRT‐PCR (Figure S6I, Supporting Information) and western blot (Figure S6F,J, Supporting Information). When knocking‐down the expression of *Fxr1*, the proliferation rate of C2C12 myoblasts decreased (Figure S6G,H, Supporting Information), and the differentiation and fusion processes were inhibited (Figure S6K,L, Supporting Information). These results revealed an impairment in myoblast proliferation and differentiation upon *Fxr1* deficiency.

Next, we transfected the overexpression vectors for *Fxr1^E15+^* and *Fxr1^E15^* ^−^ separately into the *Fxr1*‐knockdown cells (sh1‐Fxr1). Both qRT‐PCR and western blot results indicated that there was a significant restoration in the expression of differentiation markers (*MyHC*, *Myod1* and *Myog*) when overexpressing *Fxr1^E15+^* (Figure 5J; Figure S6M, Supporting Information). However, when overexpressing *Fxr1^E15^* ^−^, there was no expression change of *MyHC* and other differentiation markers (Figure 5J; Figure S6M, Supporting Information). What's more, the immunofluorescence staining results indicated that the rescue of differentiation and fusion impairment could be elicited only when the *Fxr1^E15+^* isoform was expressed, demonstrating the essential role of its specific activity in myoblast differentiation and fusion (Figure 5K,L).

These results indicate that different isoforms of *Fxr1* exhibited distinct functions: the *Fxr1^E15^* ^−^ isoform enhances myoblast proliferation, and the *Fxr1^E15+^* isoform promotes myogenic differentiation and fusion.

### 2.6 *Rbm24* Regulates the AS of *Fxr1* Exon 15

**Figure 6:**
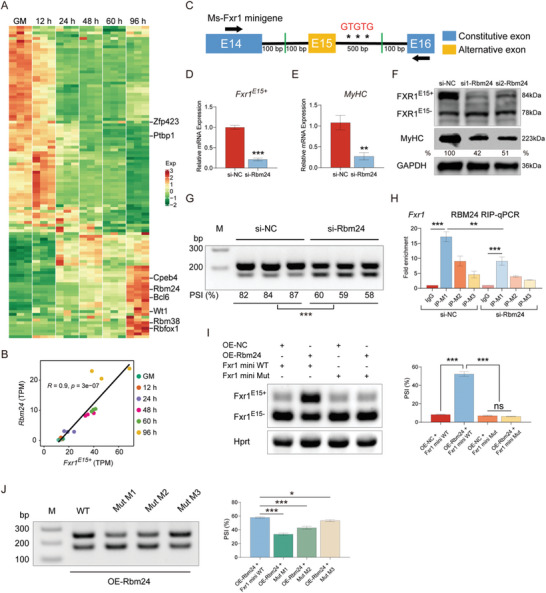
The AS of *Fxr1* exon 15 is mediated by *Rbm24*. A) Heatmap showing the expression changes of RBPs at different differentiation points (GM, 12, 24, 48, 60, and 96 h) in C2C12 myoblasts. B) The expression correlation between *Rbm24* and *Fxr1^E15+^* during C2C12 myoblasts differentiation based on the RNA‐seq data. C) Potential *Rbm24* binding motif analyses and scheme of minigene construction of *Fxr1*. Potential *Rbm24* binding sites (GTGTG, red color) were found 88 nt (M1), 311 nt (M2) and 488 nt (M3) downstream of exon 15. The semiquantitative RT‐PCR primers are indicated by black arrowheads. D, E) qRT‐PCR assay to verify the expression of *Fxr1^E15+^* (D) and *MyHC* (E) after the knockdown of *Rbm24* in C2C12 myoblasts, which were transfected with siRNAs and placed in DM for 3 days. F) Western blot analysis of FXR1 isoforms and MyHC protein expression levels after the knockdown of *Rbm24*. G) Semi‐quantitative RT‐PCR analyses of splicing changes of *Fxr1* exon 15 after *Rbm24* knocking‐down. The Percent Spliced In (PSI) values are displayed below each gel image. H) RIP‐qPCR assay showing the enrichment of *Rbm24* on IgG and *Fxr1* in the si‐NC or si‐Rbm24 treated cells. The regions harboring these three motif sites (M1‐M3) of *Rbm24*. I) Semi‐quantitative RT‐PCR analysis of *Fxr1* minigene (WT and Mut) in response to *Rbm24* overexpression. WT: GTGTG; Mut: three motif sites all mutated to ACACA. J) Semi‐quantitative RT‐PCR analysis of *Fxr1* minigene (WT, Mut M1, M2, and M3) in response to *Rbm24* overexpression. WT: GTGTG; Mut M1, M2, and M3: three motif sites each mutated individually to ACACA. The relative protein levels were normalized to those of the control GAPDH. The results are represented as the means ± SD. All data were obtained from at least three independent experiments. *P‐*values were calculated using Student's t‐test. *P < 0.05, **P < 0.01, ***P < 0.001. NC, negative control; ns indicates statistical non‐significance.

We next sought to understand the regulatory mechanism underlying the differential functions of *Fxr1* isoforms. The regulation of RNA binding proteins (RBPs) on AS in myogenesis has been reported.^[^ ^20^ ^]^ To identify RBPs that regulating the AS of *Fxr1* exon 15, we first analyzed the expression of RBPs during myoblasts differentiation. In C2C12 myoblasts, we found that 112 RBPs were differentially expressed between DM and GM phases in both pig and mouse (**Figure** **6**A; Table S7, Supporting Information), highlighting the dynamic regulation of RBPs in myogenesis. We analyzed the expression correlation between RBPs and *Fxr1^E15+^*, and predicted binding motifs in the upstream or downstream introns of *Fxr1* exon 15 by RBPmap.^[^ ^47^ ^]^ Interestingly, we found that *Rbm24*, a muscle‐specifically expressed RBP, had the similar expression pattern as the muscle‐specific inclusion of exon 15 in *Fxr1* (Figure 4C; Figure S7, Supporting Information), and was highly positively correlated with *Fxr1^E15+^* (Person's R = 0.9) (Figure 6B). Reanalysis of the skeletal muscle transcriptome of *Rbm24* conditional knockout mice suggested that *Rbm24* could affect the AS of *Fxr1* exon 15.^[^ ^18^ ^]^ Besides, there are three binding motifs (M1‐3) for *Rbm24* within the downstream intron of exon 15 (Figure 6C), suggesting *Rbm24* might target *Fxr1* to regulate the splicing of exon 15.

To explore the interaction between *Rbm24* and *Fxr1*, we first transfected siRNA targeting *Rbm24* into myoblasts and maintained induction for 72 h in DM. We found the expression of *Fxr1^E15+^* and *MyHC* was significantly decreased after *Rbm24* knocking down at both mRNA and protein levels (Figure 6D–F). Meanwhile, we observed the proportion of *Fxr1^E15+^* transcript decreased, while the proportion of *Fxr1^E15^* ^−^ transcript increased after *Rbm24* knocking down, resulting the significant decrease of the exon 15 inclusion (Figure 6G).

RIP‐qPCR assay was conducted to validate the targeting relationship between *Rbm24* and the regions harboring these motif sites. The results indicated that *Rbm24* could directly bind to the intronic region downstream of exon 15 in the *Fxr1* pre‐mRNA with a stronger binding preference with M1 (Figure 6H). Meanwhile, using 293T cells, a non‐myogenic cell line, we constructed a conventional minigene‐splicing reporter system as shown in Figure 6C. Remarkably, our results revealed that the co‐expression of *Rbm24* with *Fxr1* minigenes recapitulated the observed AS pattern. Specifically, when co‐transfecting *Rbm24* overexpression vector with *Fxr1* minigene wide‐type vector, the inclusion level of *Fxr1* exon 15 significantly increased. When the three binding sites were mutated, no significant change was observed in the inclusion level of exon 15 after *Rbm24* overexpression (Figure 6I). Furthermore, we individually mutated each of the three potential motif sites. The results showed that mutations at all three sites decreased the inclusion level of *Fxr1* exon 15, with the M1 site exhibiting the largest effect. Together with the RIP‐qPCR results (Figure 6J), we concluded that *Rbm24* preferentially binds to the M1 site. Collectively, these results indicate that *Rbm24* regulates the expression and splicing of *Fxr1* by directly binding to the downstream intron of muscle‐specific exon 15.

### 2.7 The *Fxr1^E15+^* Isoform Regulates Gene Expression and AS During Myogenesis

**Figure 7:**
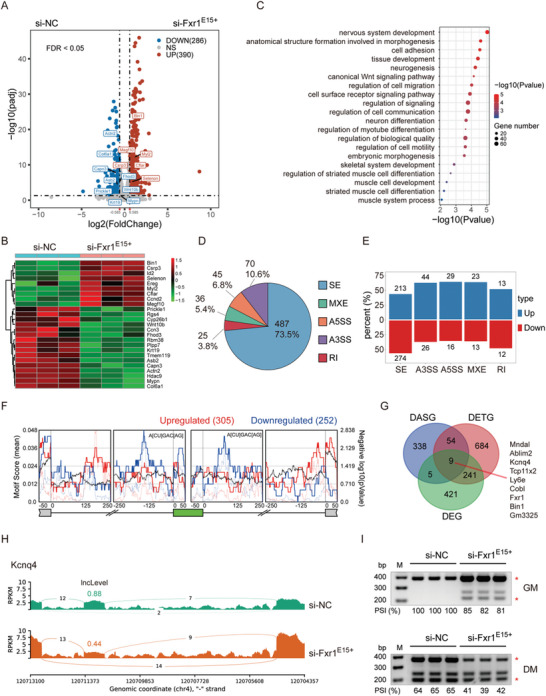
*Fxr1^E15+^* regulates gene expression and AS during myogenesis. A) Volcano plot presents the DEGs (|Fold Change| > 1.5 and FDR < 0.05) after downregulating the expression of *Fxr1^E15+^* in differentiated C2C12 myotubes. The genes associated with muscle cell development are labeled. B) Heatmap showing the expression of representative DEGs enriched in GO terms for the muscle cell development and muscle cell differentiation. C) GO enrichment analysis of downregulated genes. D) Pie charts showing the number and ratio of differential splicing events in *Fxr1^E15+^* knocked down C2C12 myotubes compared to the si‐NC cells. E) The number and percentage of *Fxr1^E15+^* modulated AS events demonstrating increased or decreased PSI upon *Fxr1^E15+^* depletion in C2C12 myotubes. F) The enrichment of canonical *Fxr1* binding motif A[CU]GAC[AG] in the upstream and downstream introns of cassette exons. The solid and dashed lines represent the motif enrichment score and the statistical significance, respectively. The green box represents the cassette exon. G) The Venn diagram showing the overlap among genes with differentially spliced SEs (DASGs; |deltaPSI| > 0.1 and FDR < 0.05), genes with differentially expressed transcripts (DETGs; |FC| > 2 and FDR < 0.05), and DEGs (|FC| > 1.5 and FDR < 0.05) after *Fxr1^E15+^* knocking‐down. H) Sashimi plot showing AS of *Kcnq4* exon 9 in C2C12 myoblasts treated with si‐NC and si‐Fxr1^E15+^. I) Semi‐quantitative RT‐PCR analyses of splicing changes of *Kcnq4* exon 9 after *Fxr1^E15+^* knocking‐down (n = 3). The Percent Spliced In (PSI) values are displayed below each gel image.

To explore the mechanism of *Fxr1* splicing regulating myogenesis, we conducted RNA‐seq analysis in differentiated C2C12 myoblasts after *Fxr1^E15+^* knocking down and found that the expression of 676 genes were significantly changed (|Fold Change| > 1.5 and FDR < 0.05), including 390 upregulated and 286 downregulated genes (**Figure** **7**A). The expression of many genes involved in muscle cell development and differentiation, such as *Bin1*, *Wnt10b*, *Capn3*, *Rgs4* and *Hacd9*, were regulated by *Fxr1^E15+^* knocking down (Figure 7B). GO analysis indicated that the downregulated genes were significantly enriched in many muscle‐related pathways such as the Wnt signaling pathway, myotube differentiation, and muscle cell differentiation (Figure 7C). We also observed 1080 transcripts in 988 genes that are significantly differentially expressed (|Fold Change| > 2 and FDR < 0.05), including 560 upregulated and 520 downregulated transcripts (Figure S8A, Supporting Information) in the *Fxr1^E15+^* knocking down cells. GO analysis revealed that genes with downregulated transcripts (DETGs), such as *Rgs4, Capn3*, *Hdac9, F2, Ablim2* and *Igf2bp1* (also DEGs), were significantly enriched in the biological process terms of muscle cell differentiation and metabolic process (Figure S8B, Supporting Information). The down‐regulation of these DEGs was further validated by qRT‐PCR assay (Figure S8C,D, Supporting Information). The above results revealed that *Fxr1^E15+^* modulated muscle cell differentiation by regulating the expression of differentiation associated genes.

We next analyzed the splicing changes after *Fxr1^E15+^* knocking‐down. Differential AS events between *Fxr1^E15+^* knock‐down and control cells were defined as *Fxr1^E15+^* modulated splicing events. We identified 663 *Fxr1^E15+^* modulated splicing events in 543 genes, most of them are SEs (73.5%), followed by A3SSs (10.6%) and A5SSs (6.8%) (Figure 7D). *Fxr1^E15+^* was more likely to repress the AS of SEs, but promote the AS of other splicing types (Figure 7E). Predicted by rMAPS (http://rmaps.cecsresearch.org/),^[^ ^48^ ^]^ we found that the canonical *Fxr1* binding motif A[CU]GAC[AG] was highly enriched in the introns flanking regulated exons regions (±250 bp) of the down‐regulated SEs. For the up‐regulated SEs, the binding motif of *Fxr1* was enriched in the downstream introns (+250 bp) of the upstream exon and the upstream introns (−250 bp) of the downstream exon (Figure 7F). The results indicated that the splicing regulation of *Fxr1^E15+^* largely depends on the relative distance between SEs and the binding site. Furthermore, we found that the expression of nine *Fxr1^E15+^* modulated differentially spliced genes (*Mndal*, *Ablim2*, *Kcnq4*, *Tcp11x2, Ly6e, Cobl, Fxr1, Bin1* and *Gm3325*) were differentially expressed at both gene and transcript levels (Figure 7G–I). However, the vast majority of genes expression differences may not be driven by changes in AS patterns (Figure 7G). These observations suggest that *Fxr1^E15+^* orchestrates gene expression and splicing events via distinct target genes or mechanisms during myogenesis.

### 2.8 *Fxr1^E15+^* Isoform Promotes Muscle Regeneration In Vivo

**Figure 8:**
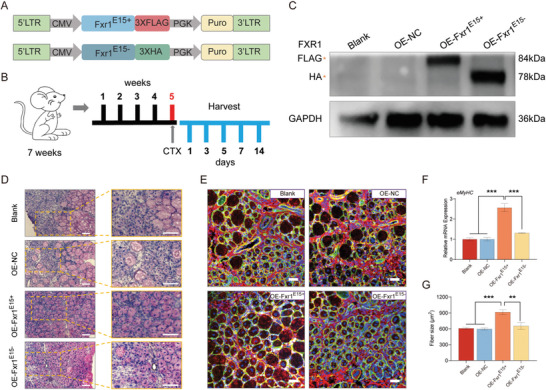
*Fxr1^E15+^* promotes muscle regeneration in vivo. A) Schematic diagram of lentiviral vectors for overexpression of *Fxr1^E15+^* and *Fxr1^E15^* ^−^, respectively. B) Schematic representation of lentivirus injection and CTX‐induced injury. Lentivirus‐mediated Fxr1‐overexpression vectors (OE‐Fxr1^E15+^ and OE‐Fxr1^E15−^) and controls (OE‐NC) were continuously injected into the tibial anterior (TA) muscle for 4 weeks and then CTX‐induced injury was performed. The TA muscle was harvested at the indicated time points following injury. C) Proteins extracted from mouse TA muscles were subjected to the western blot assay. D) H&E staining of the above injured TA muscles at 5 days post injury. Scale bar, 100 µm. E) Immunostaining of eMyHC (red), DAPI (blue) and laminin (green) of the above injured TA muscles at 5 days post injury. Scale bar, 50 µm. F) qRT‐PCR assay to verify the expression of *eMyHC* of the above injured TA muscles at 5 days post injury. G) Average area of the cross‐sections of regenerating fibres on day 5 post‐CTX injury. n = 5 per group. In (F) and (G), results are represented as the means ± SD. *P‐*values were calculated using Student's t‐test. **P < 0.01, ***P < 0.001. NC, negative control.

Given the distinct functions of the *Fxr1* isoforms in myogenesis, we next explored their functions in muscle regeneration. We first constructed the lentivirus‐mediated overexpression vectors of *Fxr1^E15+^* (OE‐Fxr1^E15+^) and *Fxr1^E15^* ^−^ (OE‐Fxr1^E15−^) and negative controls (OE‐NC) (**Figure** **8**A) and injected them in TA muscles of C57BL/6 mice (Figure 8B). As expected, the expression of these two isoforms were significantly upregulated after four weeks of injection compared to controls (Figure 8C). Subsequently, CTX (cardiotoxin) was intramuscularly injected at the same place (Figure 8B). After five days of CTX injection, H&E and immunofluorescence staining were employed to evaluate the state of muscle regeneration, we observed new myofibers containing centralized nuclei formed, as expected (Figure 8D,E). In comparison with the OE‐NC and OE‐Fxr1^E15−^ groups, OE‐Fxr1^E15+^ upregulated the expression of *eMyHC* in the TA muscle (Figure 8F). An increase in myofibers size was observed in the muscle treated with OE‐Fxr1^E15+^, whereas abundant mononucleated cells still existed in control and OE‐Fxr1^E15−^ muscles (Figure 8E,G; Figure S9, Supporting Information). The cross‐sectional area of newly formed myofibers were significantly larger in the OE‐Fxr1^E15+^ group than in the control muscle (Figure 8E,G; Figure S9, Supporting Information). Taken together, these results suggest that the *Fxr1^E15+^* isoform promotes skeletal muscle regeneration in vivo.

**Figure 9:**
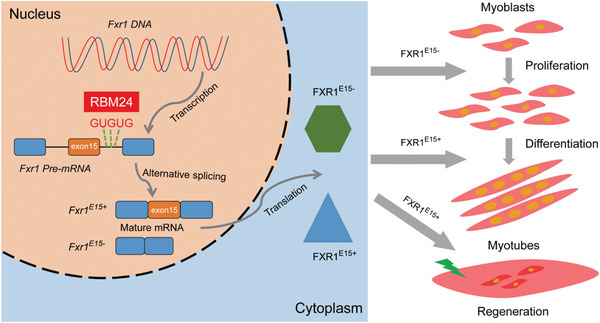
The schematic diagram illustrating the mechanism of distinct *Fxr1* isoforms regulating myogenesis and muscle regeneration. In the nucleus, *Fxr1* is transcribed into pre‐mRNA. Then, the splicing factor *RBM24* regulates the alternative splicing of *Fxr1* by binding to three GUGUG sites within the intron of *Fxr1* pre‐mRNA, generating two mature *Fxr1* transcripts, *Fxr1^E15+^* and *Fxr1^E15^* ^−^. Eventually, in the cytoplasm, the two isoforms of *Fxr1* encode two proteins. Thereby regulating different stages of myogenesis. *Fxr1^E15^* ^−^ promotes the proliferation of myoblasts, while *Fxr1^E15+^* promotes cell differentiation and facilitates muscle regeneration.

In conclusion, our findings support a model whereby *Rbm24* regulates the AS of *Fxr1*, generating two transcripts encoding different proteins. The two isoforms of *Fxr1* are dynamically expressed in different stages of myogenesis, with *Fxr1^E15^* ^−^ participating in myoblast proliferation, while *Fxr1^E15+^* regulates myogenic differentiation and muscle regeneration (**Figure** **9**).

## 3 Discussion

In previous studies, splicing transitions during myogenic differentiation have been examined.^[^ ^14^, ^49^, ^50^, ^51^ ^]^ However, due to limitations in analytical technology and sample pools, there remains significant potential for further exploration of AS events undergoing transitions. In our current investigation, we employed RNA‐seq datasets to profile the dynamic AS landscape across myogenic differentiation in five vertebrate species. Our analysis unveiled thousands of conserved DASEs between differentiation and proliferation phases. This study significantly augments the repertoire of AS events associated with myogenic differentiation. Our findings confirm that AS is a tightly regulated and evolutionarily conserved process throughout myogenic differentiation,^[^ ^14^ ^]^ emphasizing substantial gene diversification through the generation of phase‐specific isoforms. Notably, these isoforms include genes crucial for muscle cell differentiation and muscle system process, underscoring the functional significance of AS in myogenesis.

While the functional roles of microexons and NMD‐exons have been extensively investigated in the brain, their exploration in the context of muscle development and diseases remains limited.^[^ ^52^ ^]^ In our study, we identified numerous microexons and NMD‐exons that are specifically spliced during the proliferation or differentiation stages, including microexons in *Mef2d* and *Med23*, and an NMD‐exon in *Hacd1*. Notably, the splicing of the *Mef2d* microexon encodes the acidic *β*‐domain, which plays a crucial role in regulating its transcription and higher‐order assembly during myogenesis.^[^ ^53^ ^]^ Additionally, the frameshift of *Hacd1* exon 5, leading to NMD activation, contributes to the degradation of mRNA splicing isoforms. These findings underscore the significance of AS regulation as a major driver, alongside gene expression, in orchestrating myogenic differentiation through diverse regulatory mechanisms.

The developmental AS atlas across vertebrate organs and species has indicated that AS programs play disproportionate roles in brain and heart development.^[^ ^35^ ^]^ Developmentally dynamic AS has been more conserved throughout evolution compared to the more prevalent nondynamic AS.^[^ ^35^ ^]^ Global RNA‐seq analyses revealed that AS transitions are crucial for skeletal muscle function, extensive changes in gene expression and AS that occur during skeletal muscle development.^[^ ^54^, ^55^, ^56^ ^]^ AS programs are enriched in skeletal muscle,^[^ ^9^ ^]^ and the developmental transitions of AS impact skeletal muscle physiology.^[^ ^55^ ^]^ In our study, leveraging high‐resolution temporal transcriptome data in skeletal muscle,^[^ ^24^ ^]^ we uncovered over 3000 SEs that undergo dynamic splicing during skeletal muscle development. By analyzing transcriptome data from ten tissues, we pinpointed 280 SEs specifically spliced in skeletal muscle, which includes 49 skeletal muscle‐specific devSEs, such as *Fxr1* exon 15. It should be noted that the splicing levels of these DASEs in the DM phase are predominantly pronounced in skeletal muscle, whereas in the GM phase, they are widely spliced across various tissues. This pattern suggests a differentiation process wherein the gene transitions from a ubiquitous variant to a muscle‐specific isoform, potentially conferring specialized or refined properties essential for skeletal muscle function.^[^ ^57^ ^]^ This shift indicates the presence of a regulatory mechanism driving tissue‐specific splicing patterns, which could be pivotal for skeletal muscle development and differentiation. Moreover, some SE events in genes, such as *CLASP2*, are uniquely included or excluded in one tissue but display tissue‐specific expression in another. A possible explanation is that tissue‐specific splicing of these genes might lead to destabilization or degradation of their mRNA abundance in that particular tissue. However, further experiments are needed to verify this hypothesis. Further exploration is needed to understand the dynamic patterns of AS transitions across pre‐ and postnatal development of skeletal muscle in different species, specifically focusing on AS events that are specifically spliced in skeletal muscle.

The process of AS enables the generation of multiple transcripts from the same gene.^[^ ^1^ ^]^ Many myogenesis genes were reported to generate different isoforms with distinct functions and different regulatory mechanisms. For example, *Mef2D* undergoes tissue‐specific splicing, resulting in the production of a ubiquitously expressed isoform, *Mef2Da1*, and a muscle‐specific isoform, *Mef2Da2*. These two isoforms exert antagonistic effects to modulate muscle differentiation.^[^ ^58^ ^]^ Mutually exclusive splicing of the *α*1 and *α*2 exons in the *Mef2C* gene alters its functions, compared with *Mef2C* *α* *2*, *Mef2C* *α* *1* isoform interacted more strongly with and recruited *HDAC5* to repress muscle‐specific genes.^[^ ^59^ ^]^ RBP *AUF1* isoforms containing a YGG motif are competent RNA chaperones, whereas isoforms lacking the YGG motif are not.^[^ ^60^ ^]^ The two isoforms of human E‐box binding protein (*HEB*), *HEB* *α* and *HEB* *β*, have different DNA binding affinities. *HEB* *β*, but not *HEB* *α*, can complex with *MyoD*.^[^ ^61^ ^]^ In our study, we observed distinct expression patterns and functions of two *Fxr1* isoforms resulting from exon 15 splicing during myogenic differentiation: the *Fxr1^E15^* ^−^ isoform enhances myoblast proliferation, while the *Fxr1^E15+^* isoform promotes myoblast differentiation and fusion, ultimately impacting myotube formation. In vivo studies in Xenopus revealed that mis‐splicing of *Fxr1* exon 15 resulted in somite formation defects, while blocking exon 15 inclusion in vitro did not impact myotube formation.^[^ ^25^ ^]^ Importantly, in vivo experiments demonstrated that the *Fxr1^E15+^* isoform facilitates skeletal muscle regeneration. Interestingly, using our time series transcriptome data of skeletal muscle, we found the inclusion level of *Fxr1* exon 15 was continuously increased during prenatal skeletal muscle development and almost fully included after birth, suggesting the regulation of *Fxr1* exon 15 in skeletal muscle development mainly occurs before birth. These findings underscore the pivotal role of muscle‐specific splicing of *Fxr1* exon 15 in skeletal myogenesis. Furthermore, recessive mutations in exon 15 of *FXR1* can lead to congenital multi‐minicore myopathy in both humans and mice.^[^ ^26^ ^]^ Therefore, exploring the different functions of gene isoforms is significant because, for the treatment of certain diseases, partial inhibitors may provide a safer pharmacological profile compared to full inhibitors by having distinct potencies for different isoforms.^[^ ^62^ ^]^

*FXR1* AS has been previously reported to be regulated by *SRSF10* in the heart and skeletal muscle.^[^ ^63^ ^]^ Additionally, *RBM24*, a skeletal muscle‐specific splicing factor, plays a crucial role in modulating global mRNA AS.^[^ ^16^ ^]^ Knockout of *Rbm24* in mice has been shown to induce changes in the AS events of Fxr1 within skeletal muscle.^[^ ^16^, ^18^ ^]^ In our study, we identified *Rbm24* as another splicing regulator of *Fxr1* exon 15 by directly targeting its downstream intron. However, further investigation is required to elucidate whether *RBM24* independently regulates the AS of *Fxr1* exon 15 or acts in coordination with *SRSF10*. Notably, *Fxr1* exon 15 is situated within intrinsically disordered regions, which may lead to rewiring of protein interactions,^[^ ^64^, ^65^ ^]^ potentially contributing to the distinct functions of *Fxr1^E15^* ^−^ and *Fxr1^E15+^* isoforms.

The functions of RBPs in myogenesis are multifaceted. For example, *Rbfox1* can directly bind to *Mef2D* pre‐mRNA to regulate AS and promote *Mef2D* *α* *2* expression.^[^ ^66^ ^]^ In contrast, it binds to *Ntrk2* RNA, enhancing *Ntrk2* isoform expression by increasing RNA stability rather than affecting its AS.^[^ ^67^ ^]^ Additionally, *RBFOX1* can cooperate with *MBNL1* to indirectly control splicing in muscle.^[^ ^68^ ^]^ Our study reveals that *Fxr1* can also function as a splicing factor, regulating the AS of numerous genes. Interestingly, only a few genes were found to be simultaneously regulated by the *Fxr1^E15+^* isoforms at both the gene expression and splicing levels. Extensive AS transitions occur primarily without changes in gene expression,^[^ ^55^, ^69^ ^]^ which was also observed in other splicing regulators, such as *Rbfox1/2*, *Rbm24* and *SETD2*.^[^ ^18^, ^70^, ^71^ ^]^ These results suggest the existence of multiple regulatory mechanisms through which *Fxr1^E15+^* influences myogenesis. A possible explanation is that as an RBP, the distribution of *Fxr1* in the cytoplasm and nucleus leads to different functions, such as affecting mRNA stability^[^ ^27^, ^46^ ^]^ and enhancing its translation^[^ ^72^, ^73^ ^]^ in the cytoplasm.

In summary, our comprehensive profiling of AS during myogenic differentiation and skeletal muscle development offers a valuable resource for further AS studies in the skeletal muscle field. Furthermore, our findings underscore the importance of investigating protein function at the isoform level and provide a notable example of how skeletal muscle‐specific splicing regulates myogenesis.

## 4 Experimental Section

### RNA‐seq Data Collection and Analysis

RNA sequencing (RNA‐seq) data of human (PRJEB34529),^[^ ^30^ ^]^ mouse (PRJNA916831), pig (PRJNA448132),^[^ ^31^ ^]^ cattle (PRJNA725792)^[^ ^32^ ^]^ and chicken (SRR11472374)^[^ ^33^ ^]^ were downloaded from European Nucleotide Archive. The strand‐specific RNA‐seq datasets in pig skeletal muscles across 27 developmental stages of Landrace pigs (GSE157045)^[^ ^24^, ^74^, ^75^, ^76^, ^77^ ^]^ and ten tissues (skeletal muscle, subcutaneous adipose, cerebellum, cerebrum, heart, liver, lung, pancreas, small intestine, and stomach) of Duroc and Luchuan pigs (CNP0001159)^[^ ^43^ ^]^ were obtained from our previous studies. Cleaned reads were aligned to the respective reference genomes (human: GRCh38, mouse: GRCm38, pig: Sscrofa11.1, cattle: ARS‐UCD2.0, chicken: GRCg7b) using HISAT2 (v2.2.1) with default settings.^[^ ^78^ ^]^ The prepDE.py script accompanied by StringTie was utilized to count the reads of each gene or transcript. The library size of the five species can be found in Table S8 (Supporting Information). The annotation files were downloaded from Gencode for mouse (vM25) and from Ensembl for the other four species: pig (Sscrofa11.1.109), human (GRCh38.109), cattle (ARS‐UCD1.2.110), and chicken (GRCg6a.110). Gene expression was normalized with transcripts per million (TPM) by StringTie software (v1.3.6) with default settings.^[^ ^78^ ^]^ The R package DESeq2 (v1.40.2) was used to identify differential genes (|Fold Change (FC)| > 1.5 and false discovery rate (FDR) < 0.05) and transcripts (|FC| > 2 and FDR < 0.05) based on the count matrix for each of the comparisons DM over GM.^[^ ^79^ ^]^

### Gene Ontology Enrichment Analysis

Gene ontology (GO) enrichment analysis was performed with DAVID 6.8.^[^ ^80^ ^]^ Representative terms were selected with the cutoff of *P‐*values < 0.01 and visualized with ggplot2 (version 3.5.1) R package.

### Identification of Differential Alternative Splicing Events

Using the bam files generated in RNA‐seq data analysis, the different types of AS events were identified using the rMATS (v4.0.2),^[^ ^34^ ^]^ namely alternative 5’ splice site (A5SS), alternative 3’ splice site (A3SS), mutually exclusive exon (MXE), skipped exon (SE), and retained intron (RI). Because SE was the most prevalent and most well‐characterized type of AS events in mammalian transcriptomes, it focused on SE events. The quantification of each SE event was determined using exon using the percent‐spliced‐in (PSI) metric based on long (L) and short (S) forms of splicing events presents (equation shown below). Briefly, a PSI value was given according to the ratio of the long form on total form present (short form and long form) to characterize inclusion of exon.

(1) $$\mathrm{PSI}=L/\left(L+S\right)$$

Significant differential events entail filtering based on the following criteria: |deltaPSI| > 0.1 and FDR < 0.05. Differential events were visualized with rmats2sashimiplot (v2.0.4).

### Developmentally Dynamic and Tissue‐Specific Skipped Exons

After the quantification of SEs using the strand‐specific RNA‐seq datasets in pig skeletal muscle at 27 developmental stages, missing PSI values (n = 4729, 0.6% of all PSI values) were imputed using R missForest package. To ensure reliability of the imputation and downstream analyses, it only included an alternative exon in our analyses if it passed the following filters: 1) Average PSI within 0.05 and 0.95; 2) Average total read count (inclusion counts + skipping count) ≥10; 3) Number of samples with missing PSI value < 5; 4) max (PSI) – min (PSI) > 0.05. After filtering, 9071 high‐confident SEs were retained for further analysis.

After the quantification of SEs using the strand‐specific RNA‐seq dataset in ten tissues of Luchuan and Duroc pigs, PSI values were imputed using R missForest package. The mean PSI of each SE in each tissue was calculated. Tissue‐specific SEs were identified by ROKU function in R TCC package (version 1.42.0).^[^ ^81^ ^]^ The SEs with modH value less than 2 were considered as tissue‐specific SEs.

### Cell Culture

C2C12 undifferentiated cells (myoblasts) were maintained at 37 °C under 5% CO_2_ in DMEM (Gibco, USA) supplemented with 10% FBS (ExCell Bio, Uruguay), 1% penicillin/streptomycin (Gibco, USA) under low confluence conditions (< 50%). For differentiation of myoblasts into myotubes, cells were washed with PBS and then cultured in DMEM supplemented with 2% horse serum (Biological Industries, Israel), 1% penicillin/streptomycin when the cells were > 90%. 293T cells were cultured in DMEM supplemented with 10% FBS, 1% penicillin/streptomycin in a 5% CO_2_ humidified incubator at 37 °C.

### RNA Extraction and qRT‐PCR

Mouse and pig tissues, and cells were proceed as required. Total RNA was extracted using Trizol (Invitrogen, USA) following the manufacturer's suggested protocol. The quality and quantity of RNA were assessed using the NanoDrop 2000 (Thermo Fisher Scientific, USA). The cDNAs were prepared using reverse transcriptase (Vazyme, China). And then, quantitative real‐time PCR (qRT‐PCR) was conducted using Fast ChamQ Universal SYBR qPCR Master Mix (Vazyme, China) for mRNA on an ABI Step One Plus Real‐Time PCR system (Applied Biosystems, USA). The 2^−ΔΔCt^ method was employed to analyze the relative expression levels of mRNA. GAPDH was used as endogenous controls to normalize the expression mRNA. The primers sequences (Sangon Biotech, China) were listed in Table S9 (Supporting Information).

### Semiquantitative RT‐PCR Analysis of AS Events

The primer sequences of semi‐quantitative RT‐PCR (Sangon Biotech, China) were listed in Table S9 (Supporting Information). 25–30 cycles for amplification were used with an annealing temperature of 58 °C (adjusted according to the primers). PCR products were separated by 2.5% agarose gel in 1×TAE buffer for 40–60 min at 120 V. And the 15% polyacrylamide gel were used to separate the stage‐specific microexons in 0.5×TBE buffer, first for 30 min at 60 V, followed by an additional 90 min at 120 V. Quantification of gels was performed by densitometry using ImageJ software (National Institutes of Health).

### Plasmids Construction

Restriction enzymes (AgeI and EcoRI for pLKO.1, BamHI and XhoI for pCMV‐3×FALG, pCMV‐3×HA, pLVX‐3×FALG and pLVX‐3×HA) were from Thermo Fisher Scientific, and competent cells were from TIANGEN. Oligonucleotides used for plasmid generation were synthesized at Sangon Biotech. DNA Ligation Kit and ClonExpress II One Step Cloning Kit used for pLKO.1‐shFxr1, pCMV‐Fxr1^E15+^‐3×FALG, pCMV‐Fxr1^E15−^‐3×HA, pLVX‐Fxr1^E15+^‐3×FALG and pLVX‐Fxr1^E15−^‐3×HA were purchased from Takara Bio and Vazyme respectively. Sequencing was also performed by Sangon Biotech. The primers sequences were listed in Table S9 (Supporting Information).

### Cell Line Construction

The transfer plasmid pLKO.1‐shFxr1 and two plasmids from the second‐generation lentiviral packaging system, pMD2.G (Envelope Plasmid) and psPAX2 (Packaging Plasmid), were co‐transfected into 293T cells for lentivirus packaging. After 60 h, the lentivirus supernatant was harvested. Subsequently, undifferentiated C2C12 cells were infected with the pLKO.1‐shFxr1 lentivirus and 8 µg ml^−1^ polybrene (TR‐1003, Sigma–Aldrich, USA). After 72 h of transduction, cells were selected using 1.5 µg ml^−1^ puromycin (ant‐pr‐1, InvivoGen, France) for 4 days. *Fxr1* knockdown C2C12 cells were then collected and labeled as shFxr1‐C2C12 for rescue experiments.

### Transfection of siRNA or Plasmids

For exploring cellular proliferation function, C2C12 cells were seeded into six‐well plates and transfected (≈40% confluent) with 2 µL siRNAs or 1 µg plasmids using Lipofectamine 3000 (Invitrogen, USA) following the manufacturer's protocols. Before reaching a cell density of 80%, qRT‐PCR, western blot and EdU assay were conducted. Additionally, C2C12 and shFxr1‐C2C12 cells were seeded into six‐well plates for one day, and the next day, transfections (≈80% confluent) were performed using Lipofectamine 3000 (Invitrogen, USA). Each well was transfected 5 µL siRNAs or 3 µg plasmids. After transfection for 12 h, the differentiation medium was used for myoblasts differentiation induced for 3 days, and then, qRT‐PCR, western blot and immunofluorescence assay were conducted. siRNA oligonucleotides against siFxr1‐exon 15, si‐Rbm24 as well as the control siRNA‐NC, were obtained from RiboBio (China). siRNA sequences were provided in Table S10 (Supporting Information).

### Cell Proliferation Assays

Cell proliferation capacity was measured by EdU staining using the BeyoClickTM EdU Cell Proliferation Kit (Beyotime, China). Briefly, the cells were fixed in 4% paraformaldehyde and permeabilized with 0.5% Triton X‐100. Next, cells were stained with Click Additive Solution in the dark for 30 min, and the nuclei were counterstained with DAPI solution. Images were acquired using a Nikon ECLIPSE Ti microscope, and the ImageJ software was employed to calculate the proportion of EdU‐positive cells.

### Immunofluorescence Staining

Cells were fixed in a solution of 4% paraformaldehyde in 1 phosphate‐buffered saline (PBS, pH 7.4) for 20 min, washed with PBS and incubated for 15 min in a solution of 0.2% Triton X‐100 and 5% Bovine Serum Albumin (BSA) in PBS. Fixed cells were then incubated in the same solution containing primary antibody at 4 °C for overnight (16 h), washed with PBS and incubated for 2 h at room temperature in PBS containing secondary antibody. Immunofluorescence staining primary antibodies included MyHC (MF20; 2 µg ml^−1^; Developmental Studies Hybridoma Bank, USA), and a secondary antibody (anti‐mouse CY3 and anti‐mouse FITC, Servicebio, China). DAPI was used to visualize the cell nuclei with a fluorescence microscope. Differentiation index (percentage of nuclei in MyHC‐positive myotubes/total nuclei) and fusion index (percentage of nuclei in MyHC‐positive myotubes with ≥ 2 nuclei/total nuclei) were calculated.

### Protein Extraction and Western Blot Analysis

Proteins were extracted by RIPA buffer (Thermo Fisher Scientific, USA) supplemented with protease inhibitor (Roche, Switzerland). The concentration of obtained protein was measured by the BCA kit (Beyotime, China). The proteins were separated in 8% and 10% sodium dodecyl sulfate‐polyacrylamide gel electrophoresis (SDS‐PAGE) gels (EpiZyme, USA) and transferred onto 0.45 µm Hybridization Nitrocellulose Filter (NC) membrane (Merck, USA), and then probed with antibodies following standard procedures.

The following antibodies were used in the present work: GAPDH (60004‐1‐Ig, 1:50000, Proteintech Group, China), Fxr1 (13194‐1‐AP, 1:5000, Proteintech Group, China), Ki67 (ab16667; 1:1000; Abcam, UK), PCNA (1:5000, 10205‐2‐AP, Proteintech Group, China), MyHC (MF20; 0.2 µg ml^−1^; Developmental Studies Hybridoma Bank, USA), DYKDDDDK tag Polyclonal antibody (20543‐1‐AP, 1:20000, Proteintech Group, China) and HA tag Polyclonal antibody (51064‐2‐AP, 1:5000, Proteintech Group, China). Secondary antibodies: Goat Anti‐Rabbit (ZB‐2301, 1:1000, ZSGB‐BIO, China) and Goat Anti‐Mouse (ZB‐2305, 1:1000, ZSGB‐BIO, China).

### RNA Immunoprecipitation Assay

RNA immunoprecipitation (RIP) was performed as previously described. Briefly, 20 µL of the DYKDDDDK Tag (FLAG) antibody (#14793, Cell Signaling Technology, USA), and IgG antibody were incubated with 60 µL of protein A‐Sepharose slurry beads (washed and equilibrated in cell lysis buffer) for 4 h at 4 °C. Beads were washed three times with cell lysis buffer and incubated with 500 µg of cell extracts over‐night at 4 °C. Beads were then washed again three times with cell lysis buffer and co‐immunoprecipitated RNA was then eluted and processed for qRT‐PCR analysis. The primer sequences (Sangon Biotech, China) were listed in Table S9 (Supporting Information).

### Muscle Injury and Regeneration

All animal procedures followed protocols approved by the Hubei Province of China for Biological Studies Animal Care and Use Committee, the Chinese Academy of Agricultural Sciences and the Institutional Animal Care and Use Committee. Mice were anesthetized throughout modeling using isoflurane. Prior to inducing injury, the hind limb was disinfected using 75% alcohol. Then, muscle injury was induced in 7‐weeks‐old male mice by injecting 50 µL of 50 µM CTX (cardiotoxin, 217503, Sigma–Aldrich, USA) in PBS into the TA muscle. Muscles were then harvested at the indicated days after injection to assess the regeneration and repair. The detailed steps to examine skeletal muscle injury are available in this protocol.^[^ ^82^ ^]^

### Histology Staining

TA muscles were dissected from mice, embedded in the OCT compound (Tissue‐Tek; 4583, SAKURA, USA) and snap‐frozen by immersing in liquid nitrogen. Using a cryostat (LEICA CM1950, Germany), 15 µm cryosections were prepared. Haematoxylin and eosin staining of muscle sections was performed according to a previously reported method.^[^ ^83^ ^]^ The cross‐sectional areas of individual myofibres were quantified using ImageJ software. Immunohistochemical staining was performed as described previously^[^ ^83^ ^]^ and visualized using a confocal laser scanning microscope (Nikon Corporation, Japan). For immunofluorescence staining, the cryosections were permeabilized using 0.5% Triton X‐100 (Sigma, China) for 30 min and then blocked with 5% bovine serum albumin (BSA) (Biofroxx, Germany) for 1 h. The primary antibody eMyHC (F1.652, 3 µg ml^−1^, Developmental Studies Hybridoma Bank, USA) and Laminin (ab11575, 1:500, Abcam, UK) were incubated at room temperature for 2 h. And the secondary antibody (anti‐mouse CY3 and anti‐rabbit FITC, 1:500, Servicebio, China) incubated at room temperature for 1 h. Nuclei were labeled with DAPI (Beyotime, China) for 10 min.

### Statistical Analysis

For wet experiments, the results were represented as the means ± SD. *P‐*values were calculated using Student's t‐test. Statistical significance was set at *P < 0.05, **P < 0.01 and ***P < 0.001.

## Conflict of Interest

The authors declare no conflict of interest.

## Author Contributions

W.W., X.F., and W.L. contributed equally to this work. Z.T. and Y.Y. conceptualized the study. W.W., Y.Y., and Z.T. designed experiments. Y.Y. and X.F. analyzed and interpreted the data. W.W., W.L., and Y.H. performed the experiments. W.W. and Y.Y. wrote the manuscript. W.W., Y.Y., S.Z., and Z.T. reviewed and edited the manuscript. Y.Y., Z.T., and S.Z. supervised the study. Z.T. and Y.Y. acquired funding for the study.

## Supporting information



Supporting Information

## Data Availability

The data that support the findings of this study are available in the supplementary material of this article.

## References

[1] L. E. Marasco , A. R. Kornblihtt , Nat. Rev. Mol. Cell Biol. 2023, 24, 242. 36229538 10.1038/s41580-022-00545-z

[2] D. O. Bates , J. C. Morris , S. Oltean , L. F. Donaldson , C. J. Garland , Pharmacol. Rev. 2017, 69, 63. 28034912 10.1124/pr.115.011239 PMC5226212

[3] N. A. Patel , C. E. Chalfant , J. E. Watson , J. R. Wyatt , N. M. Dean , D. C. Eichler , D. R. Cooper , J. Biol. Chem. 2001, 276, 22648. 11283022 10.1074/jbc.M101260200

[4] D. Li , W. Yu , M. Lai , Acta Pharm. Sin. B 2023, 13, 3181. 37655328 10.1016/j.apsb.2023.05.022 PMC10465970

[5] Y. Xu , W. Wu , Q. Han , Y. Wang , C. Li , P. Zhang , H. Xu , Open Biol. 2019, 9, 180239. 30836866 10.1098/rsob.180239 PMC6451366

[6] T. Fei , Y. Chen , T. Xiao , W. Li , L. Cato , P. Zhang , M. B. Cotter , M. Bowden , R. T. Lis , S. G. Zhao , Q. Wu , F. Y. Feng , M. Loda , H. H. He , X. S. Liu , M. Brown , Proc. Natl. Acad. Sci. USA 2017, 114, E5207. 28611215 10.1073/pnas.1617467114 PMC5495225

[7] S. Choi , N. Cho , K. K. Kim , Exp. Mol. Med. 2023, 55, 755. 37009804 10.1038/s12276-023-00981-7 PMC10167241

[8] K. Nakka , C. Ghigna , D. Gabellini , F. J. Dilworth , Skeletal Muscle 2018, 8, 8. 29510724 10.1186/s13395-018-0152-3 PMC5840707

[9] J. C. Castle , C. Zhang , J. K. Shah , A. V. Kulkarni , A. Kalsotra , T. A. Cooper , J. M. Johnson , Nat. Genet. 2008, 40, 1416. 18978788 10.1038/ng.264 PMC3197713

[10] T. Braun , M. Gautel , Nat. Rev. Mol. Cell Biol. 2011, 12, 349. 21602905 10.1038/nrm3118

[11] C. F. Bentzinger , Y. X. Wang , M. A. Rudnicki , Cold Spring Harbor Perspect. Biol. 2012, 4, a008342. 10.1101/cshperspect.a008342 PMC3281568 22300977

[12] J. M. Hernandez‐Hernandez , E. G. Garcia‐Gonzalez , C. E. Brun , M. A. Rudnicki , Semin. Cell Dev. Biol. 2017, 72, 10. 29127045 10.1016/j.semcdb.2017.11.010 PMC5723221

[13] M. Yamamoto , N. P. Legendre , A. A. Biswas , A. Lawton , S. Yamamoto , S. Tajbakhsh , G. Kardon , D. J. Goldhamer , Stem Cell Rep. 2018, 10, 956. 10.1016/j.stemcr.2018.01.027 PMC5918368 29478898

[14] C. S. Bland , E. T. Wang , A. Vu , M. P. David , J. C. Castle , J. M. Johnson , C. B. Burge , T. A. Cooper , Nucleic Acids Res. 2010, 38, 7651. 20634200 10.1093/nar/gkq614 PMC2995044

[15] R. Grifone , X. Xie , A. Bourgeois , A. Saquet , D. Duprez , D.‐L. Shi , Mech. Dev. 2014, 134, 1. 25217815 10.1016/j.mod.2014.08.003

[16] J. Yang , L. H. Hung , T. Licht , S. Kostin , M. Looso , E. Khrameeva , A. Bindereif , A. Schneider , T. Braun , Dev. Cell 2014, 31, 87. 25313962 10.1016/j.devcel.2014.08.025

[17] T. Zhang , Y. Lin , J. Liu , Z. G. Zhang , W. Fu , L. Y. Guo , L. Pan , X. Kong , M. K. Zhang , Y. H. Lu , Z. R. Huang , Q. Xie , W. H. Li , X. Q. Xu , Stem Cells 2016, 34, 1776. 26990106 10.1002/stem.2366

[18] M. Zhang , Y. Han , J. Liu , L. Liu , L. Zheng , Y. Chen , R. Xia , D. Yao , X. Cai , X. Xu , Theranostics 2020, 10, 11159. 33042276 10.7150/thno.44389 PMC7532667

[19] P. Montanes‐Agudo , S. Aufiero , E. N. Schepers , I. van der Made , L. Cocera‐Ortega , A. C. Ernault , S. Richard , D. W. D. Kuster , V. M. Christoffels , Y. M. Pinto , E. E. Creemers , Cardiovasc. Res. 2023, 119, 1161. 36627242 10.1093/cvr/cvad007 PMC10202634

[20] R. K. Singh , Z. Xia , C. S. Bland , A. Kalsotra , M. A. Scavuzzo , T. Curk , J. Ule , W. Li , T. A. Cooper , Mol. Cell 2014, 55, 592. 25087874 10.1016/j.molcel.2014.06.035 PMC4142074

[21] J. Cao , S. K. Verma , E. Jaworski , S. Mohan , C. K. Nagasawa , K. Rayavara , A. Sooter , S. N. Miller , R. J. Holcomb , M. J. Powell , P. Ji , N. D. Elrod , E. Yildirim , E. J. Wagner , V. Popov , N. J. Garg , A. L. Routh , M. N. Kuyumcu‐Martinez , Cell Rep. 2021, 37, 109910. 34731606 10.1016/j.celrep.2021.109910 PMC8600936

[22] L. Andersson , Y. Yang , J. Yan , X. Fan , J. Chen , Z. Wang , X. Liu , G. Yi , Y. Liu , Y. Niu , L. Zhang , L. Wang , S. Li , K. Li , Z. Tang , PLoS Genet. 2021, 17, e1009910. 34780471 10.1371/journal.pgen.1009910 PMC8629385

[23] R. A. Tumasian , A. Harish , G. Kundu , J.‐H. Yang , C. Ubaida‐Mohien , M. Gonzalez‐Freire , M. Kaileh , L. M. Zukley , C. W. Chia , A. Lyashkov , W. H. Wood , Y. Piao , C. Coletta , J. Ding , M. Gorospe , R. Sen , S. De , L. Ferrucci , Nat. Commun. 2021, 12, 2014. 33795677 10.1038/s41467-021-22168-2 PMC8016876

[24] Y. Yang , X. Fan , J. Yan , M. Chen , M. Zhu , Y. Tang , S. Liu , Z. Tang , Nucleic Acids Res. 2021, 49, 1313. 33434283 10.1093/nar/gkaa1203 PMC7897484

[25] J. A. Smith , E. G. Curry , R. E. Blue , C. Roden , S. E. R. Dundon , A. Rodríguez‐Vargas , D. C. Jordan , X. Chen , S. M. Lyons , J. Crutchley , P. Anderson , M. E. Horb , A. S. Gladfelter , J. Giudice , J. Cell Biol. 2020, 219, e201911129. 32328638 10.1083/jcb.201911129 PMC7147106

[26] M. C. Estan , E. Fernandez‐Nunez , M. S. Zaki , M. I. Esteban , S. Donkervoort , C. Hawkins , J. A. Caparros‐Martin , D. Saade , Y. Hu , V. Bolduc , K. R. Chao , J. Nevado , A. Lamuedra , R. Largo , G. Herrero‐Beaumont , J. Regadera , C. Hernandez‐Chico , E. F. Tizzano , V. Martinez‐Glez , J. J. Carvajal , R. Zong , D. L. Nelson , G. A. Otaify , S. Temtamy , M. Aglan , M. Issa , C. G. Bonnemann , P. Lapunzina , G. Yoon , V. L. Ruiz‐Perez , Nat. Commun. 2019, 10, 797. 30770808 10.1038/s41467-019-08548-9 PMC6377633

[27] A. B. Herman , C. N. Vrakas , M. Ray , S. E. Kelemen , M. J. Sweredoski , A. Moradian , D. S. Haines , M. V. Autieri , Cell Rep. 2018, 24, 1176. 30067974 10.1016/j.celrep.2018.07.002 PMC11004729

[28] A. S. Paul , C. Corbett , A. Peluzzo , S. Kelemen , R. Okune , D. S. Haines , K. Preston , S. Eguchi , M. V. Autieri , Cell Rep. 2023, 42, 112381. 37043351 10.1016/j.celrep.2023.112381 PMC10564969

[29] M.‐E. Huot , N. Bisson , L. Davidovic , R. Mazroui , Y. Labelle , T. Moss , E. W. Khandjian , Mol. Biol. Cell 2005, 16, 4350. 16000371 10.1091/mbc.E05-04-0304 PMC1196343

[30] V. Mournetas , E. Massouridès , J. B. Dupont , E. Kornobis , H. Polvèche , M. Jarrige , A. R. L. Dorval , M. R. F. Gosselin , A. Manousopoulou , S. D. Garbis , D. C. Górecki , C. Pinset , J. Cachexia, sarcopenia Muscle 2021, 12, 209. 33586340 10.1002/jcsm.12665 PMC7890274

[31] T.‐N. Tran‐Thi , S. Wang , A. A. Adetula , C. Zou , A. I. Omar , J.‐L. Han , D.‐X. Zhang , S.‐H. Zhao , Gene 2019, 695, 113. 30633943 10.1016/j.gene.2018.12.059

[32] X. Yang , J. Wang , X. Ma , J. Du , C. Mei , L. Zan , Front. Cell Develop. Biol. 2021, 9, 785380. 10.3389/fcell.2021.785380 PMC8685427 34938736

[33] W. Luo , Z. Lin , J. Chen , G. Chen , S. Zhang , M. Liu , H. Li , D. He , S. Liang , Q. Luo , D. Zhang , Q. Nie , X. Zhang , J. Cachexia, Sarcopenia Muscle 2021, 12, 1704. 34427057 10.1002/jcsm.12767 PMC8718073

[34] S. Shen , J. W. Park , Z. X. Lu , L. Lin , M. D. Henry , Y. N. Wu , Q. Zhou , Y. Xing , Proc. Natl. Acad. Sci. USA 2014, 111, E5593. 25480548 10.1073/pnas.1419161111 PMC4280593

[35] P. V. Mazin , P. Khaitovich , M. Cardoso‐Moreira , H. Kaessmann , Nat. Genet. 2021, 53, 925. 33941934 10.1038/s41588-021-00851-w PMC8187152

[36] J. Blondelle , Y. Ohno , V. Gache , S. Guyot , S. Storck , N. Blanchard‐Gutton , I. Barthélémy , G. Walmsley , A. Rahier , S. Gadin , M. Maurer , L. Guillaud , A. Prola , A. Ferry , G. Aubin‐Houzelstein , J. Demarquoy , F. Relaix , R. J. Piercy , S. Blot , A. Kihara , L. Tiret , F. Pilot‐Storck , J. Mol. Cell Biol. 2015, 7, 429. 26160855 10.1093/jmcb/mjv049 PMC4589950

[37] S. Han , C. Cui , H. He , X. Shen , Y. Chen , Y. Wang , D. Li , Q. Zhu , H. Yin , Int. J. Mol. Sci. 2019, 20, 5130. 31623157 10.3390/ijms20205130 PMC6829482

[38] R. Kjøbsted , J. L. W. Roll , N. O. Jørgensen , J. B. Birk , M. Foretz , B. Viollet , A. Chadt , H. Al‐Hasani , J. F. P. Wojtaszewski , Diabetes 2019, 68, 1427. 31010958 10.2337/db19-0050

[39] L. Yang , L.‐L. Chen , Cell 2014, 159, 1488. 25525868 10.1016/j.cell.2014.12.004

[40] R. Faraway , J. Ule , Nat. Ecol. Evolution 2019, 3, 526. 10.1038/s41559-019-0818-1 30833756

[41] B. Choudhary , O. Marx , A. D. Norris , Cell Rep. 2021, 36, 109464. 34348142 10.1016/j.celrep.2021.109464 PMC8378409

[42] J.‐w. Yin , Y. Liang , J. Y. Park , D. Chen , X. Yao , Q. Xiao , Z. Liu , B. Jiang , Y. Fu , M. Bao , Y. Huang , Y. Liu , J. Yan , M.‐s. Zhu , Z. Yang , P. Gao , B. Tian , D. Li , G. Wang , Genes Dev. 2012, 26, 2192. 22972934 10.1101/gad.192666.112 PMC3465740

[43] Y. Liu , Y. Fu , Y. Yang , G. Yi , J. Lian , B. Xie , Y. Yao , M. Chen , Y. Niu , L. Liu , Genetics Selection Evolution 2022, 54, 62. 10.1186/s12711-022-00754-2 PMC9476355 36104777

[44] I. Letunic , P. Bork , Nucleic Acids Res. 2018, 46, D493. 29040681 10.1093/nar/gkx922 PMC5753352

[45] I. Letunic , S. Khedkar , P. Bork , Nucleic Acids Res. 2021, 49, D458. 33104802 10.1093/nar/gkaa937 PMC7778883

[46] G. A. Cox , L. Davidovic , N. Durand , O. Khalfallah , R. Tabet , P. Barbry , B. Mari , S. Sacconi , H. Moine , B. Bardoni , PLoS Genet. 2013, 9, e1003367. 23555284 10.1371/journal.pgen.1003367 PMC3605292

[47] I. Paz , I. Kosti , M. Ares , M. Cline , Y. Mandel‐Gutfreund , Nucleic Acids Res. 2014, 42, W361. 24829458 10.1093/nar/gku406 PMC4086114

[48] J. W. Park , J. Bok , E. C. Rouchka , T. L. Kook , S. Jung , J. Y. Hwang , Nucleic Acids Res. 2020, 48, W300. 32286627 10.1093/nar/gkaa237 PMC7319468

[49] C. Trapnell , B. A. Williams , G. Pertea , A. Mortazavi , G. Kwan , M. J. van Baren , S. L. Salzberg , B. J. Wold , L. Pachter , Nat. Biotechnol. 2010, 28, 511. 20436464 10.1038/nbt.1621 PMC3146043

[50] J. R. Wheeler , O. N. Whitney , T. O. Vogler , E. D. Nguyen , B. Pawlikowski , E. Lester , A. Cutler , T. Elston , N. Dalla Betta , K. R. Parker , K. E. Yost , H. Vogel , T. A. Rando , H. Y. Chang , A. M. Johnson , R. Parker , B. B. Olwin , eLife 2022, 11, e75844. 35695839 10.7554/eLife.75844 PMC9191894

[51] E. T. Wang , A. J. Ward , J. M. Cherone , J. Giudice , T. T. Wang , D. J. Treacy , N. J. Lambert , P. Freese , T. Saxena , T. A. Cooper , C. B. Burge , Genome Res. 2015, 25, 858. 25883322 10.1101/gr.184390.114 PMC4448682

[52] P. Benoist , J. A. Mas , R. Marco , M. Cervera , J. Biol. Chem. 1998, 273, 7538. 9516455 10.1074/jbc.273.13.7538

[53] M. Gonczi , J. M. C. Teixeira , S. Barrera‐Vilarmau , L. Mediani , F. Antoniani , T. M. Nagy , K. Feher , Z. Raduly , V. Ambrus , J. Tozser , E. Barta , K. E. Kover , L. Csernoch , S. Carra , M. Fuxreiter , Nat. Commun. 2023, 14, 1329. 36898987 10.1038/s41467-023-37017-7 PMC10006080

[54] M. S. Penna , R. C. Hu , G. G. Rodney , T. A. Cooper , Life Sci. Alliance 2023, 6, e202201868. 36977593 10.26508/lsa.202201868 PMC10052820

[55] A. E. Brinegar , Z. Xia , J. A. Loehr , W. Li , G. G. Rodney , T. A. Cooper , eLife 2017, 6, e27192. 28826478 10.7554/eLife.27192 PMC5577920

[56] P. Luo , Z. Wang , C. Su , H. Li , H. Zhang , Y. Huang , W. Chen , Poultry Sci. 2023, 102, 102403. 36584419 10.1016/j.psj.2022.102403 PMC9827075

[57] E. T. Wang , R. Sandberg , S. Luo , I. Khrebtukova , L. Zhang , C. Mayr , S. F. Kingsmore , G. P. Schroth , C. B. Burge , Nature 2008, 456, 470. 18978772 10.1038/nature07509 PMC2593745

[58] S. Sebastian , H. Faralli , Z. Yao , P. Rakopoulos , C. Palii , Y. Cao , K. Singh , Q. C. Liu , A. Chu , A. Aziz , M. Brand , S. J. Tapscott , F. J. Dilworth , Genes Dev. 2013, 27, 1247. 23723416 10.1101/gad.215400.113 PMC3690398

[59] M. Zhang , B. Zhu , J. Davie , J. Biol. Chem. 2015, 290, 310. 25404735 10.1074/jbc.M114.606277 PMC4281734

[60] L. Sanger , J. Bender , K. Rostowski , R. Golbik , H. Lilie , C. Schmidt , S. E. Behrens , S. Friedrich , RNA Biol. 2021, 18, 843. 32924750 10.1080/15476286.2020.1822637 PMC8163432

[61] M. H. Parker , R. L. Perry , M. C. Fauteux , C. A. Berkes , M. A. Rudnicki , Mol. Cell. Biol. 2006, 26, 5771. 16847330 10.1128/MCB.02404-05 PMC1592768

[62] M. E. Gurney , A. B. Burgin , O. T. Magnusson , L. J. Stewart , Handbook Exp. Pharmacol. 2011, 204, 167. 10.1007/978-3-642-17969-3_7 21695640

[63] N. Wei , Y. Cheng , Z. Wang , Y. Liu , C. Luo , L. Liu , L. Chen , Z. Xie , Y. Lu , Y. Feng , Cell Rep. 2015, 13, 1647. 26586428 10.1016/j.celrep.2015.10.038

[64] M. Buljan , G. Chalancon , A. K. Dunker , A. Bateman , S. Balaji , M. Fuxreiter , M. M. Babu , Curr. Opin. Struct. Biol. 2013, 23, 443. 23706950 10.1016/j.sbi.2013.03.006

[65] A. Patil , A. R. Strom , J. A. Paulo , C. K. Collings , K. M. Ruff , M. K. Shinn , A. Sankar , K. S. Cervantes , T. Wauer , J. D. St Laurent , G. Xu , L. A. Becker , S. P. Gygi , R. V. Pappu , C. P. Brangwynne , C. Kadoch , Cell 2023, 186, 4936. 37788668 10.1016/j.cell.2023.08.032 PMC10792396

[66] V. Runfola , S. Sebastian , F. J. Dilworth , D. Gabellini , J. Cell Sci. 2015, 128, 631. 25609712 10.1242/jcs.161059 PMC4357529

[67] F. Tomassoni‐Ardori , G. Fulgenzi , J. Becker , C. A. Barrick , M. E. Palko , S. Kuhn , V. N. Kopardé , M. Cam , S. Yanpallewar , S. Oberdoerffer , L. Tessarollo , eLife 2019, 8, e49673. 31429825 10.7554/eLife.49673 PMC6715404

[68] A. L. de Munain , R. Klinck , A. Fourrier , P. Thibault , J. Toutant , M. Durand , E. Lapointe , M.‐L. Caillet‐Boudin , N. Sergeant , G. Gourdon , G. Meola , D. Furling , J. Puymirat , B. Chabot , PLoS One 2014, 9, e107324. 25211016 10.1371/journal.pone.0107324 PMC4161394

[69] J. Giudice , Z. Xia , E. T. Wang , M. A. Scavuzzo , A. J. Ward , A. Kalsotra , W. Wang , X. H. Wehrens , C. B. Burge , W. Li , T. A. Cooper , Nat. Commun. 2014, 5, 3603. 24752171 10.1038/ncomms4603 PMC4018662

[70] R. K. Singh , A. M. Kolonin , M. L. Fiorotto , T. A. Cooper , Cell Rep. 2018, 24, 197. 29972780 10.1016/j.celrep.2018.06.017 PMC6070147

[71] H. J. Wiedner , E. V. Torres , R. E. Blue , Y. H. Tsai , J. Parker , J. Giudice , FEBS J. 2022, 289, 6799. 35724320 10.1111/febs.16553 PMC9796740

[72] J. George , Y. Li , I. P. Kadamberi , D. Parashar , S.‐W. Tsaih , P. Gupta , A. Geethadevi , C. Chen , C. Ghosh , Y. Sun , S. Mittal , R. Ramchandran , H. Rui , G. Lopez‐Berestein , C. Rodriguez‐Aguayo , G. Leone , J. S. Rader , A. K. Sood , M. Dey , S. Pradeep , P. Chaluvally‐Raghavan , Cell Rep. 2021, 37, 109934. 34731628 10.1016/j.celrep.2021.109934 PMC8675433

[73] J.‐Y. Kang , Z. Wen , D. Pan , Y. Zhang , Q. Li , A. Zhong , X. Yu , Y.‐C. Wu , Y. Chen , X. Zhang , P.‐C. Kou , J. Geng , Y.‐Y. Wang , M.‐M. Hua , R. Zong , B. Li , H.‐J. Shi , D. Li , X.‐D. Fu , J. Li , D. L. Nelson , X. Guo , Y. Zhou , L.‐T. Gou , Y. Huang , M.‐F. Liu , Science 2022, 377, 6607. 10.1126/science.abj6647 35951695

[74] Y. Yang , M. Zhu , X. Fan , Y. Yao , J. Yan , Y. Tang , S. Liu , K. Li , Z. Tang , DNA Res. 2019, 26, 261. 31231762 10.1093/dnares/dsz006 PMC6589548

[75] J. Yan , Y. Yang , X. Fan , G. Liang , Z. Wang , J. Li , L. Wang , Y. Chen , A. A. Adetula , Y. Tang , K. Li , D. Wang , Z. Tang , J. Cachexia, Sarcopenia Muscle 2022, 13, 696. 34811940 10.1002/jcsm.12859 PMC8818660

[76] A. A. Adetula , X. Fan , Y. Zhang , Y. Yao , J. Yan , M. Chen , Y. Tang , Y. Liu , G. Yi , K. Li , Z. Tang , RNA Biol. 2021, 18, 439. 34314293 10.1080/15476286.2021.1954380 PMC8677025

[77] W. Wang , W. Li , W. Liu , Z. Wang , B. Xie , X. Yang , Z. Tang , Genes 2024, 15, 196. 38397185 10.3390/genes15020196 PMC10888101

[78] M. Perțea , D. Kim , G. Pertea , J. T. Leek , S. L. Salzberg , Nat. Protoc. 2016, 11, 1650. 27560171 10.1038/nprot.2016.095 PMC5032908

[79] M. I. Love , W. Huber , S. Anders , Genome Biol. 2014, 15, 550. 25516281 10.1186/s13059-014-0550-8 PMC4302049

[80] B. T. Sherman , M. Hao , J. Qiu , X. Jiao , M. W. Baseler , H. C. Lane , T. Imamichi , W. Chang , Nucleic Acids Res. 2022, 50, W216. 35325185 10.1093/nar/gkac194 PMC9252805

[81] K. Kadota , J. Ye , Y. Nakai , T. Terada , K. Shimizu , BMC Bioinformatics 2006, 7, 294. 16764735 10.1186/1471-2105-7-294 PMC1501047

[82] G. A. Garry , M. L. Antony , D. J. Garry , Meth. Mol. Biol. 2016, 1460, 61. 10.1007/978-1-4939-3810-0_6 27492166

[83] C. Wang , F. Yue , S. Kuang , Bio‐protocol 2017, 7, e2279. 28752107 10.21769/BioProtoc.2279 PMC5526362

