# Supplementary material for: The Spatial‐Temporal Alternative Splicing Profile Reveals the Functional Diversity of FXR1 Isoforms in Myogenesis
Source: Adv Sci (Weinh). 2024 Nov 5;11(47):2405157. doi: 10.1002/advs.202405157 (PMC11653684; doi:10.1002/advs.202405157)
Supplement: Supplementary file 1 — Supporting Information [file ADVS-11-2405157-s001.pdf]

ADVANCED SCIENCE
Open Access

Supporting Information

for *Adv. Sci.*, DOI 10.1002/advs.202405157

The Spatial-Temporal Alternative Splicing Profile Reveals the Functional Diversity of FXR1 Isoforms in Myogenesis

Wei Wang, Xinhao Fan, Weiwei Liu, Yuxin Huang, Shuhong Zhao, Yalan Yang and Zhonglin Tang**

Supporting Information

The Spatial-Temporal Alternative Splicing Profile Reveals the Functional Diversity of FXR1 Isoforms in Myogenesis

Wei Wang,† Xinhao Fan,† Weiwei Liu,† Yuxin Huang, Shuhong Zhao, Yalan Yang, and Zhonglin Tang**

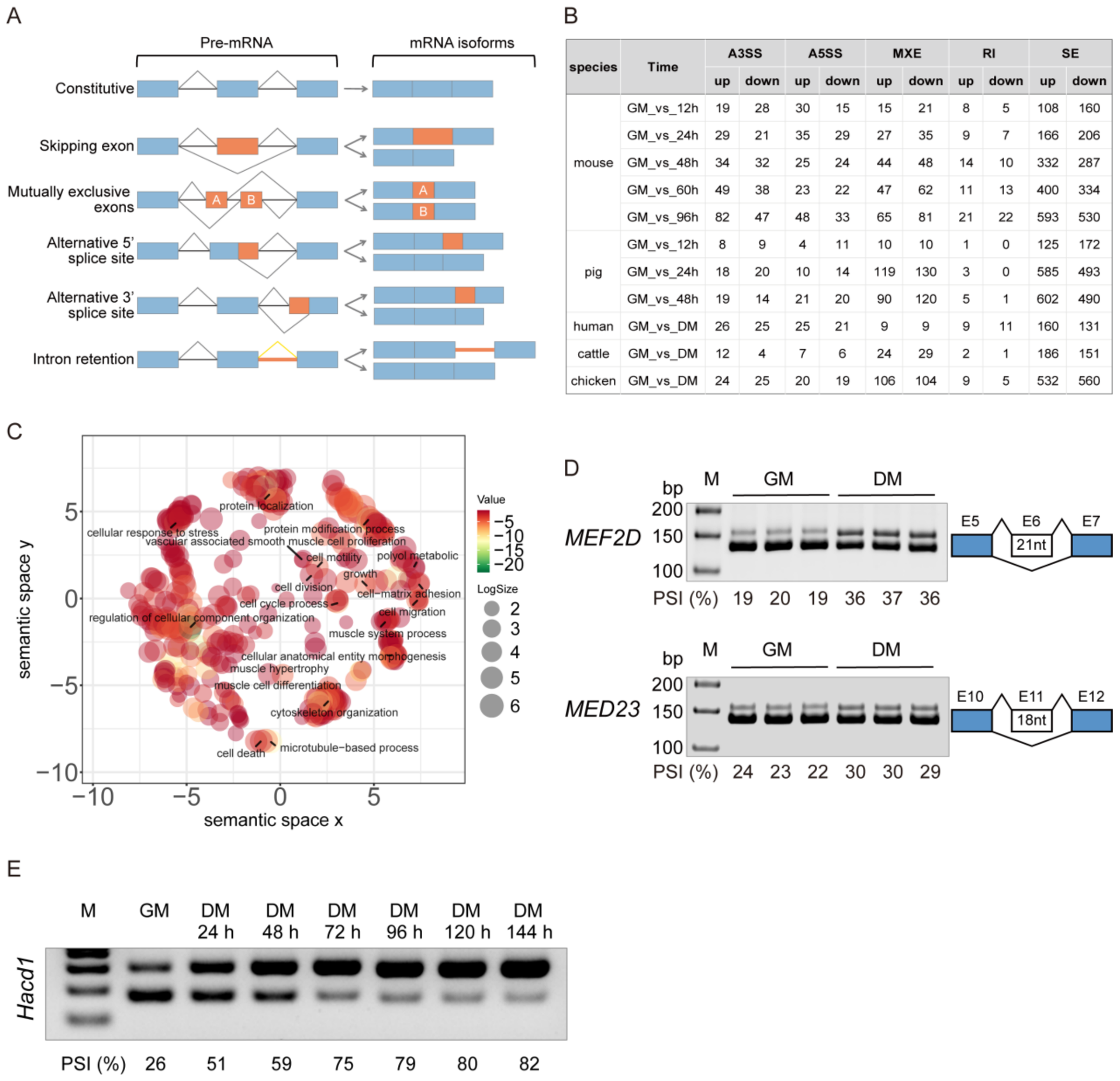

species	Time	A3SS		A5SS		MXE		RI		SE	
		up	down	up	down	up	down	up	down	up	down
mouse	GM_vs_12h	19	28	30	15	15	21	8	5	108	160
	GM_vs_24h	29	21	35	29	27	35	9	7	166	206
	GM_vs_48h	34	32	25	24	44	48	14	10	332	287
	GM_vs_60h	49	38	23	22	47	62	11	13	400	334
	GM_vs_96h	82	47	48	33	65	81	21	22	593	530
pig	GM_vs_12h	8	9	4	11	10	10	1	0	125	172
	GM_vs_24h	18	20	10	14	119	130	3	0	585	493
	GM_vs_48h	19	14	21	20	90	120	5	1	602	490
human	GM_vs_DM	26	25	25	21	9	9	9	11	160	131
cattle	GM_vs_DM	12	4	7	6	24	29	2	1	186	151
chicken	GM_vs_DM	24	25	20	19	106	104	9	5	532	560

Figure S1. Differentially spliced events between the proliferation and differentiation stages of myoblasts in human, mouse, pig, cattle and chicken. A) Constitutive splicing (top) and five different AS types. B) The number of differential splicing events between DM and GM phases in mouse, pig, human, cattle and chicken. C) The scatter plot shows the GO enrichment analysis of genes with DASEs. REVIGO (http://revigo.irb.hr/) was used to remove redundancy in GO terms. Some muscle development-related GO terms were significantly enriched, such as muscle cell differentiation, muscle hypertrophy, muscle system process and smooth muscle cell proliferation. D) Semiquantitative RT-PCR analyses of splicing changes of two microexons between DM and GM phases in pig

skeletal muscle satellite cells. E) Semiquantitative RT-PCR analyses of splicing changes of *Hacd1* NMD-SE during C2C12 myoblasts proliferation (GM) and differentiation (DM; 24 h, 48 h, 72 h, 96 h, 120 h and 144 h).

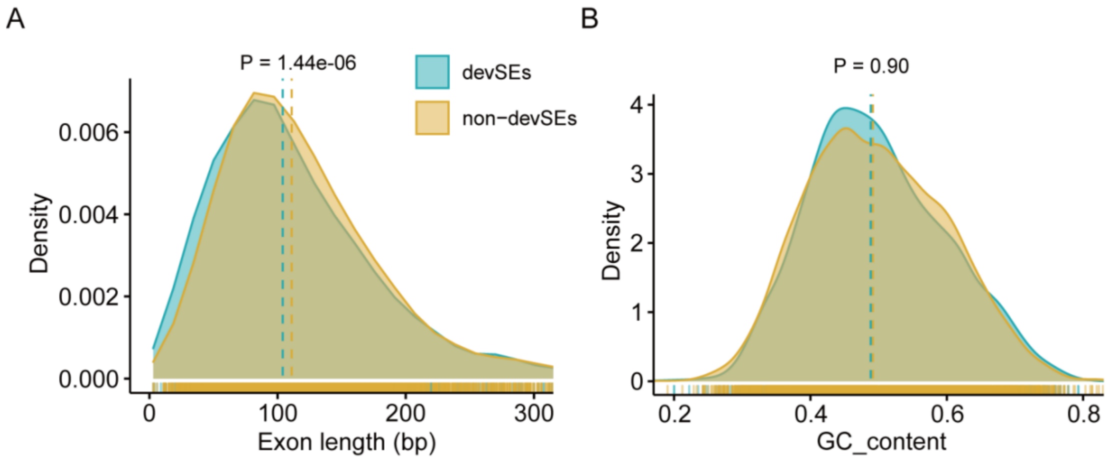

Figure S2. The comparisons between devSEs and non-devSEs on exon length (A) and GC content (B).

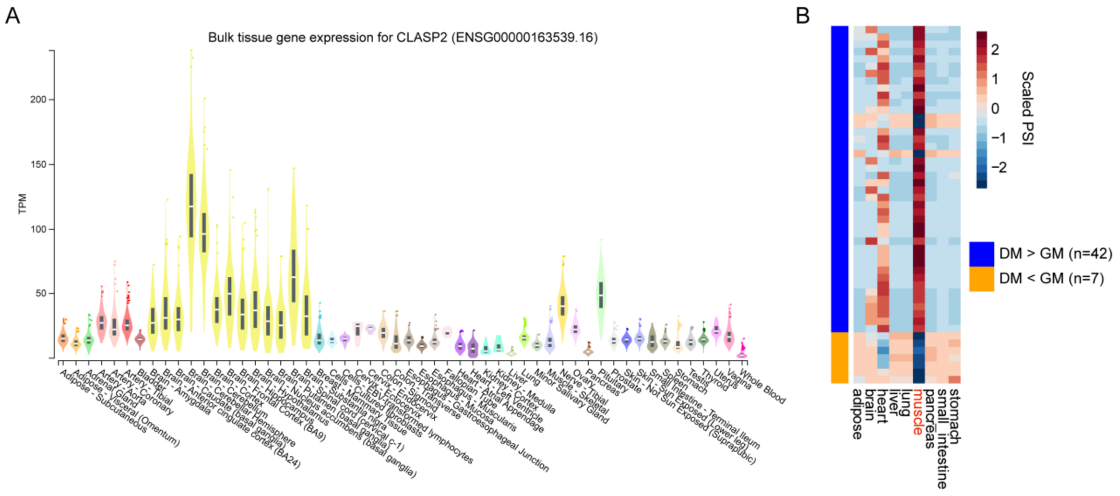

Figure S3. Expression profile of CLASP2 and splicing patterns of 49 DASEs. A) The expression of *CLASP2* in different tissues of human. Data from the GTEx (https://www.gtexportal.org/home/gene/CLASP2). B) The splicing pattern of 49 skeletal muscle specific and development associated DASEs across various tissues.

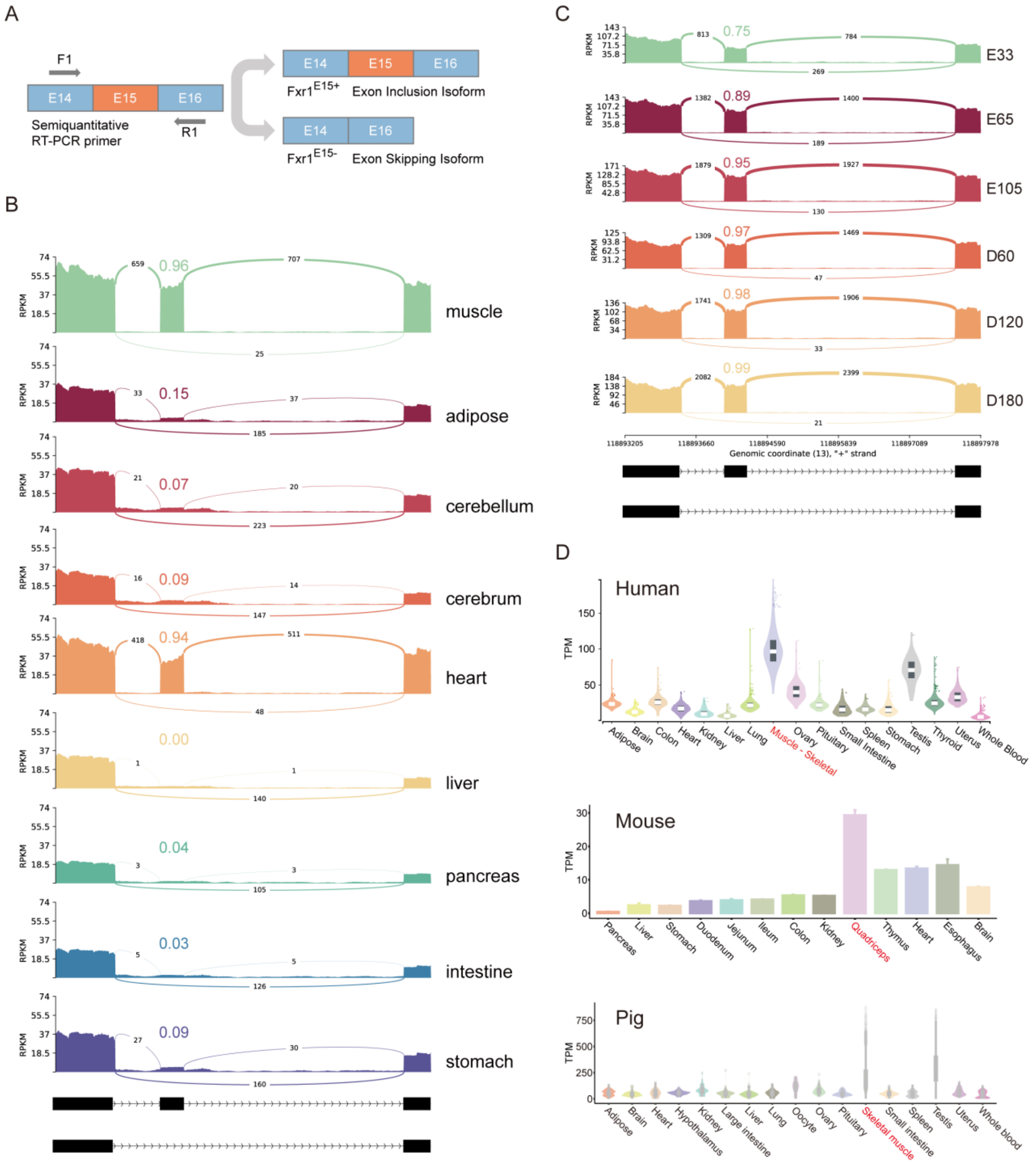

Figure S4. The *Fxr1* gene undergoes specific AS in skeletal muscle. A) The positions of semiquantitative primers employed to investigate the extent of AS of *Fxr1*. B) Sashimi plot showing AS of *Fxr1* exon 15 in different tissues of pigs. C) Sashimi plot showing AS of *Fxr1* exon 15 during skeletal muscle development in pigs. D) The expression level of *Fxr1* gene in different tissues of human (GTEx data), mouse (https://www.ebi.ac.uk/biostudies/arrayexpress/studies/E-MTAB-6081) and pig (https://pigome.com).

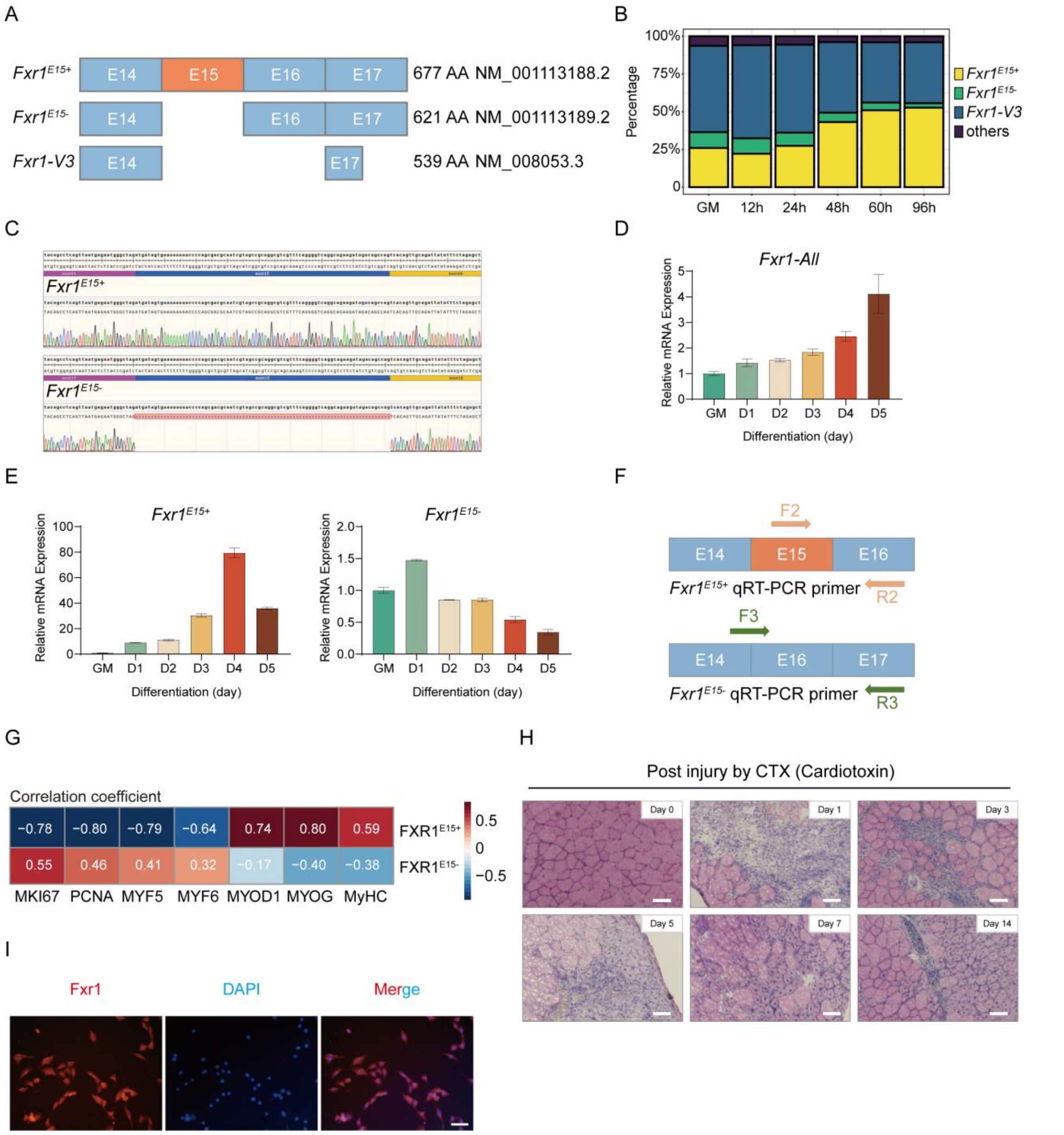

Figure S5. Features of *Fxr1* and its three major isoforms. A) Location of the alternatively spliced region within mouse *Fxr1*. $Fxr1^{E15+}$ is the longest isoform; $Fxr1^{E15-}$ lacks alternate in-frame segments in both the mid-coding and 3' coding regions, compared to variant 1, resulting in a shorter protein; Fxr1-V3, this variant 3 differs in the 3' UTR, and lacks an alternate in-frame segment in the mid-coding region and two alternate exons in the 3' coding region, compared to variant 1. The resulting protein (isoform 3, also known as isoform a) contains a distinct C-terminus and is shorter than isoform 1. B) Transcriptome sequencing data quantified the percentage of expression levels for $Fxr1^{E15+}$, $Fxr1^{E15-}$, *Fxr1-V3*, and other transcripts at during differentiation of C2C12 myoblasts. C) Alignment of $Fxr1^{E15+}$ and $Fxr1^{E15-}$ sequences after Sanger sequencing. D) qRT-PCR assay to verify the expression trend of *Fxr1-All* (all transcripts) mRNA during C2C12 myoblast differentiation. E) qRT-PCR assay to verify the expression trend of $Fxr1^{E15+}$ and $Fxr1^{E15-}$ mRNA during C2C12 myoblast differentiation. The results are represented as the means ± SD. F) The design of qRT-PCR primers for detecting the expression of $Fxr1^{E15+}$ and $Fxr1^{E15-}$ isoforms. G) Heatmap showing the expression correlation of $FXR1^{E15+}$ and $FXR1^{E15-}$ with proliferation and differentiation markers based on the RNA-seq data in porcine skeletal muscle satellite cells. The values and color represent the correlation coefficient. H) H&E staining of TA muscles at different time points post injury by

CTX in C57BL/6J mice. I) Immunofluorescence analysis of FXR1 (red) subcellular distribution in C2C12 myoblasts. Nuclei were counterstained with DAPI (blue) and merge images are shown in the right panel. Scale bar, 100 μm.

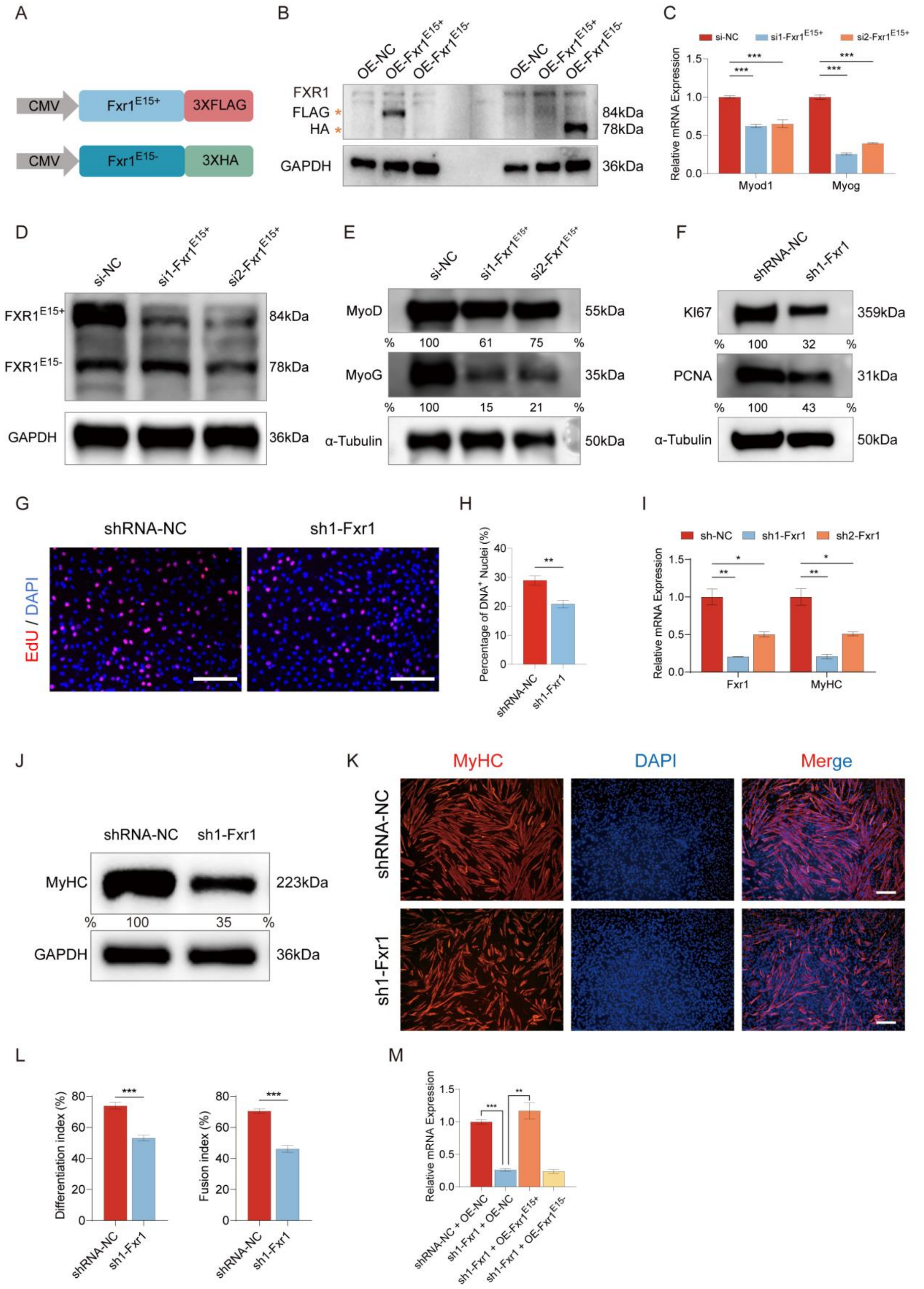

Figure S6. Efficiency validation of the Fxr1 gene and its two isoforms by knockdown and overexpression and and their corresponding functions in myogenesis. A) Schematic diagram of vectors for overexpression of $Fxr1^{E15+}$ and $Fxr1^{E15-}$. B) Proteins extracted from C2C12 myoblasts were subjected to the western blot assay. The plasmids OE-

NC, OE-Fxr1^{E15+}, and OE-Fxr1^{E15-} were individually transfected into C2C12 myoblasts. C) qRT-PCR analysis of *Myod1* and *Myog* mRNA expression after knocking-down *Fxr1*$^{E15+}$. D) Western blot analysis of two isoforms of FXR1 protein expression levels after knockdown of *Fxr1* exon 15. E) Western blot analysis of MyoD and MyoG protein expression levels after knockdown of Fxr1 exon 15. F) Western blot analysis of Ki67 and PCNA protein expression in the indicated Fxr1 knockdown. G) Cells undergoing DNA replication were stained by EdU (red) and cell nuclei were stained with DAPI (blue). Scale bar, 200 μm. H) Percentage of EdU$^+$ nuclei were quantitated with ImageJ software. I) Myoblasts were transfected with sh-NC (Ctrl shRNA), sh1-Fxr1 and sh2-Fxr1, 12 h later, they were placed in differentiation medium and were collected at 3 days. qRT-PCR analysis of the mRNA levels of Fxr1 and myogenic marker (MyHC) as indicated. J) Western blot analysis of MyHC protein expression in the indicated Fxr1 knockdown. K) Immunofluorescence staining of C2C12 myoblasts differentiated for 3 days. Myotubes were labeled with MyHC (red) and cell nuclei were counterstained with DAPI (blue). Scale bar, 200 μm. L) Differentiation index and fusion index of Figure S6K were quantitated with ImageJ software. M) qRT-PCR analysis of MyHC mRNA expression levels after transfection of Fxr1^{E15+} and Fxr1^{E15-} overexpression plasmids into the FXR1-knockdown cell line. The relative protein levels were normalized to those of the control GAPDH. The results are represented as the means ± SD. All data were obtained from three independent experiments. *P*-values were calculated using Student's t-test. **P* < 0.05, ***P* < 0.01, ****P* < 0.001. NC, negative control.

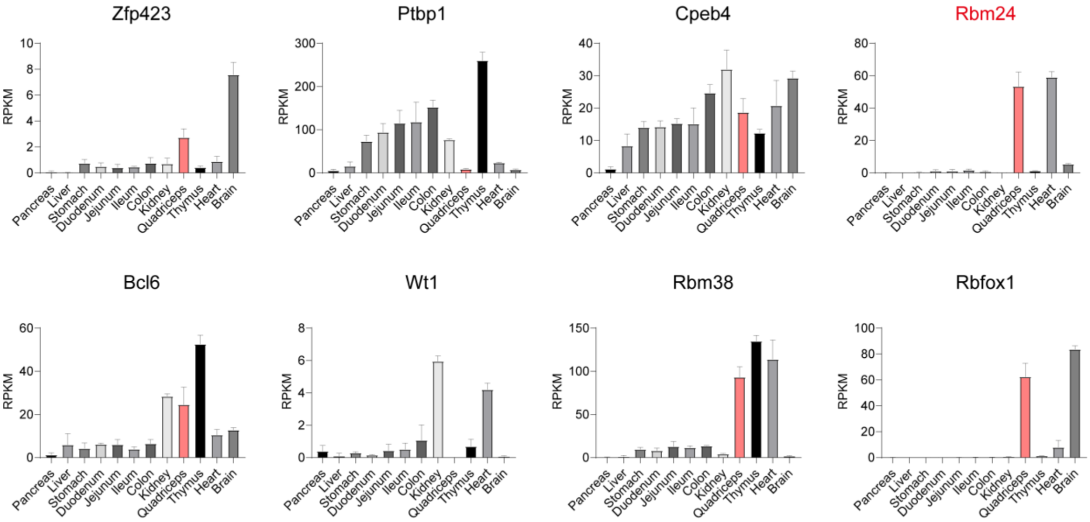

Figure S7. The expression levels of RBPs in different tissues of mouse. Rbm24 is specifically expressed in skeletal muscle and heart. This data[80] is sourced from https://www.ebi.ac.uk/biostudies/arrayexpress/studies/E-MTAB-6081#processed-data

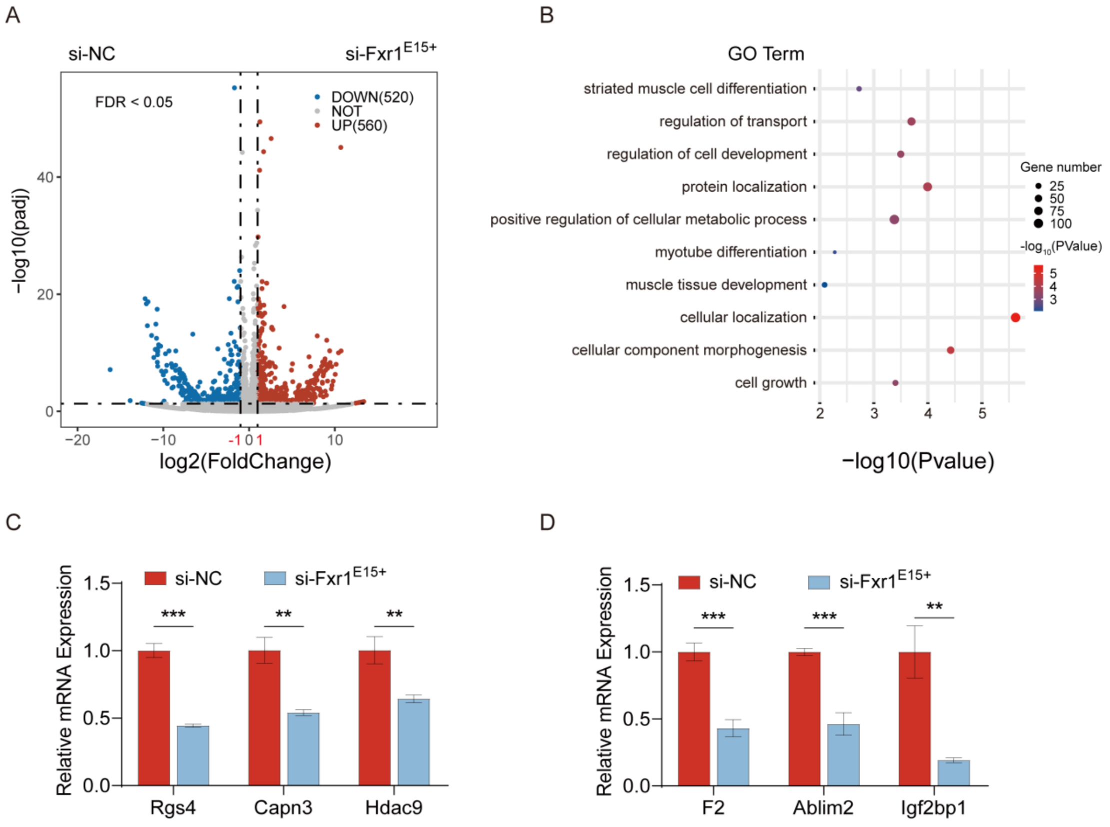

Figure S8. Differentially expressed transcripts and differentially expressed genes affected by *Fxr1*$^{E15+}$. A) Volcano plot presents the differentially expressed transcripts (|log2 Fold Change| > 1 and FDR < 0.05) after downregulating the expression of *Fxr1*$^{E15+}$ in differentiated C2C12 myotubes. B) GO enrichment analysis of genes with downregulated transcripts. Gene ontology (GO) enrichment analysis was performed with DAVID 6.8. Representative terms were selected with the cutoff of *P*-values < 0.01 and visualized with ggplot2 (version 3.5.1) R package. C, D) qRT-PCR assay to verify differentially expressed genes related to muscle cell differentiation (C) and metabolic process (D) after the *Fxr1*$^{E15+}$ knockdown. The results are represented as the means ± SD. All data were obtained from three independent experiments. *P*-values were calculated using Student's t-test. ***P* < 0.01, ****P* < 0.001.

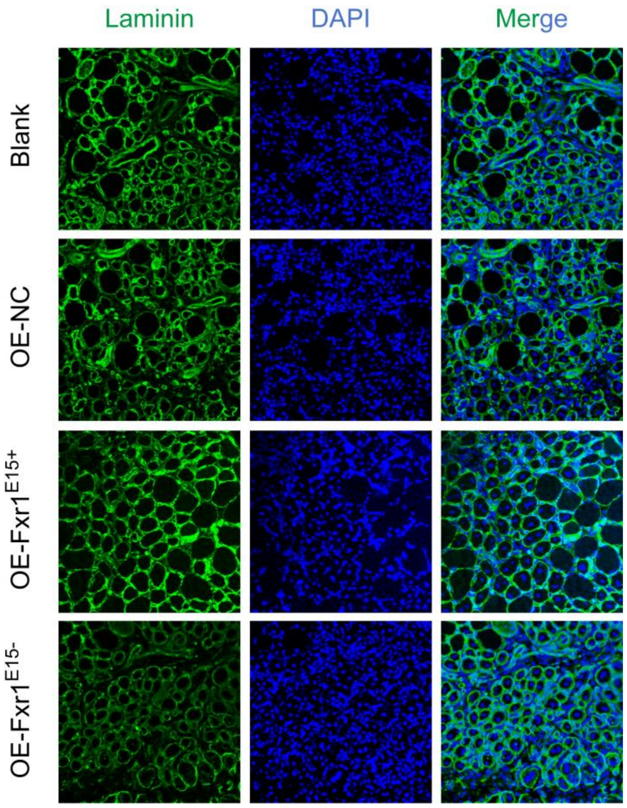

Figure S9. Single-channel images for the laminin and DAPI channels in Figure 8E.
